# Adherence to digital pregnancy care – lessons learned from the SMART start feasibility study

**DOI:** 10.1038/s41746-025-01966-8

**Published:** 2025-08-30

**Authors:** Katharina M. Jaeger, Michael Nissen, Heike Leutheuser, Nina Danzberger, Adriana Titzmann, Constanza A. Pontones, Chloë Goossens, Philipp Ziegler, Sabrina Uhrig, Lothar Haeberle, Hannah Bleher, Kristina Kast, Johannes Kornhuber, Oliver Schoeffski, Matthias Braun, Peter A. Fasching, Matthias W. Beckmann, Bjoern M. Eskofier, Hanna Huebner

**Affiliations:** 1 Machine Learning and Data Analytics Lab, Friedrich-Alexander-Universität Erlangen–Nürnberg, Erlangen, Germany; 2 Ambient Assisted Living & Medical Assistance Systems, Department of Computer Science, University of Bayreuth, Bayreuth, Germany; 3 Department of Obstetrics and Gynecology, Uniklinikum Erlangen, Friedrich-Alexander-Universität Erlangen-Nürnberg, Erlangen, Germany; 4 Department of Social Ethics, University of Bonn, Bonn, Germany; 5 Department of Healthcare Management, Friedrich-Alexander-Universität Erlangen–Nürnberg, Erlangen, Germany; 6 Department of Psychiatry and Psychotherapy, Friedrich-Alexander-Universität Erlangen–Nürnberg, Erlangen, Germany; 7 Translational Digital Health Group, Institute of AI for Health, Helmholtz Zentrum München—German Research Center for Environmental Health, Neuherberg, Germany

**Keywords:** Medical research, Health care, Health services

## Abstract

The World Health Organization increasingly highlights the role of digital health technologies in supporting prenatal care. Despite this potential, the real-world implementation of such technologies remains limited, even in high-income countries with established analog systems. We developed a comprehensive digital pregnancy care framework, SMART Start and evaluated it in a prospective study involving 528 pregnant individuals in Germany. This study is registered at the German Clinical Trials Register (DRKS00036867). Participants were equipped with a mobile app and self-examination technologies. The mobile app featured study functionality, pregnancy-related questionnaires, digital maternity records, and pregnancy-supportive content. Self-examination technologies included a standard care kit for home measurements of routine prenatal care parameters (weight, blood pressure, urinalysis), and an innovative kit with novel sensors (smartwatch, sleep analyzer). Here, we analyzed the adherence to digital pregnancy care and present the lessons learned from a clinical and technical perspective. Among all participants, 49% engaged with at least one digital package. Weekly weight tracking reached adherence rates up to 67% in the first 14 weeks. Adherence to blood pressure and urinalysis measurements was lower, peaking at 20 and 28%, respectively, but remained stable over time. Questionnaire completion rates varied in dependence on their length and complexity. 31% of users disengaged at the time of registration. While overall retention time did not significantly differ across participant subgroups (all *p* > 0.05), adherence analyses revealed meaningful group-level differences in engagement with specific self-examination protocols. This discrepancy underscores that continued participation does not necessarily imply consistent engagement with all components of the digital care model. The adherence to the study schedule demonstrated that pregnant individuals are generally willing and capable of engaging in home-based, multimodal self-monitoring; however, the importance of adaptive scheduling, patient-centered feedback, agile development, and interdisciplinary collaboration should be addressed by future studies. The presented SMART Start framework offers a pathway towards data-driven, personalized pregnancy care while potentially reducing the demand for conventional healthcare infrastructure.

## Introduction

The World Health Organization (WHO) has increasingly recognized digital health technologies for their potential to support pregnancy care by increasing access, supporting knowledge transfer, and strengthening empowerment^1^. Despite this potential, real-world adoption remains limited, even in high-income countries with established analog systems^2^. Germany, for example, ranks 16th out of 17 countries in regard to digitalization in healthcare, as indicated in a recent study by the Bertelsmann Stiftung^3^. This shows significant delays in healthcare digitalization compared to other nations. Although new legislation was introduced in 2024 to accelerate the use of electronic patient records and e-prescriptions, implementation remains fragmented, and adoption remains low^4^. These shortcomings are also evident in prenatal care, which remains largely reliant on traditional, paper-based methods. One example is the German paper-based maternity record, which is a central component of pregnancy care^5^. While the maternity record provides valuable documentation for emergency situations and subsequent pregnancies, its predominant paper format poses challenges like limited space, being prone to loss, and incomplete documentation. An electronic version has been introduced as part of the German eHealth infrastructure, but is not yet widely adopted in routine care^6^. The growing healthcare provider shortages, particularly in rural areas in Germany, make regular in-person prenatal visits challenging^7^. In Germany, routine prenatal care includes check-ups every four weeks until the 32nd week of gestation and every two weeks thereafter, following standardized maternity guidelines^8^. Many routine screening parameters, such as blood pressure and weight, are simple and cost-effective to measure at home, allowing pregnant individuals to perform these assessments themselves^9^. The integration of telehealth technologies, enabling, e.g., home-based self-monitoring for routine care, may optimize antenatal care experiences, reduce healthcare costs, and maintain maternal and neonatal health outcomes^10^.

The growing acceptance of digital health tools presents a great opportunity to further modernize prenatal care^11^. Over 50% of pregnant women in middle-to-high income countries use mobile health (mHealth) apps to track their health, manage appointments, and receive guidance^12–14^. Digital health tools, including apps and wearable devices, are particularly valued for their ease of use and the instant feedback they provide, making them an increasingly popular choice in prenatal care^15^. Some studies have explored their application within antenatal care^9^, highlighting the potential of wearables and other digital tools to use large-scale pregnancy datasets for outcome prediction and to track trajectories of biomedical signals and symptoms, offering more personalized and data-driven approaches to pregnancy care^16,17^. Fitness trackers can accurately record heart rate in pregnancy-related activities, further demonstrating their utility in monitoring maternal health^18^. Expert panels recognize digital health as an important tool that should be integrated into health systems for understanding and reducing maternal mortality^19^. However, they suggest that mobile health apps should be developed in cooperation with healthcare professionals and in line with guidelines and evidence^20,21^.

Large data sets have the potential to reveal new findings in medicine. Recent research showed that large datasets captured with smart wearable devices reveal clear trajectories of pregnancy from cycling to conception through the postpartum recovery phase, including the visualization of non-viable pregnancies^16^. Erickson et al. demonstrated the ability to predict labor onset and gestational age using physiological parameters like heart rate and sleep captured with wearable devices^22^.

Despite these advances, evidence on the use of wearable sensors in pregnancy care remains limited^23^ and a digital framework that combines routine prenatal care aspects with innovative self-monitoring technologies is still missing. Furthermore, little is known about adherence to home-based measurements and large-scale digital pregnancy studies, including compliance with various measurement protocols, engagement with app-based health tracking, and long-term participation in multifaceted digital prenatal care interventions. The adherence in virtual studies is often limited, particularly when participants receive little feedback or when monitoring protocols are not sufficiently tailored to individual needs^24^. Varying levels of technical affinity among users can hinder consistent engagement with digital tools, highlighting the importance of intuitive design and ongoing support^25^. In addition, concerns about data privacy and the handling of sensitive health data remain a major barrier to long-term participation^26^.

To address these gaps, a multidisciplinary research initiative—the SMART start study—was initiated by experts in women’s health, psychiatry and psychotherapy, computer science, ethics, health economics, clinical trials, and digital health to evaluate a novel and comprehensive digital health framework for home-based pregnancy care. The following analysis presents data on adherence patterns to interventions within a digital pregnancy care concept and identifies key barriers and facilitators of sustained engagement. Practical lessons learned during implementation offer guidance for future digital health initiatives in maternal care. This main study was supplemented by several companion studies and pre-trials, which are referenced where applicable. It represents a significant step toward modernizing pregnancy care through digital innovation.

## Results

### Participant characteristics

**Fig. 1:**
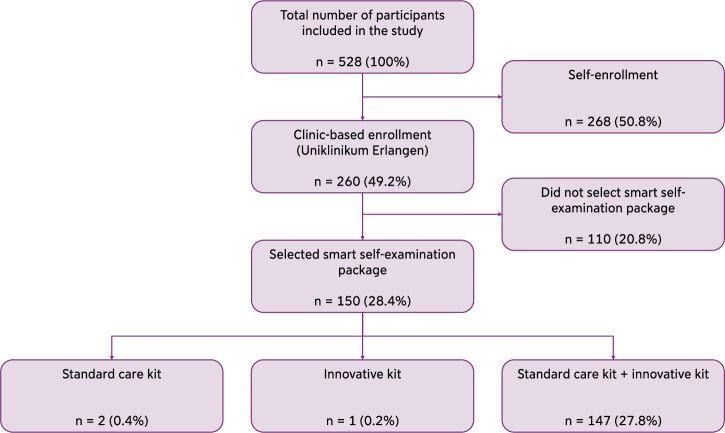
Overview of study participation possibilities and participant distribution. Absolute participant counts are shown with relative proportions in parentheses. Participants enrolled either via the university hospital (clinic-based enrolled) or independently (self-enrollment). Clinic-enrolled participants automatically received access to the digital maternity record and could opt for smart self-examination packages.

A total of 528 pregnant individuals were included in the SMART start study, of whom 260 (49.2%) were enrolled via the Uniklinikum Erlangen (UKER), see Fig. 1. Clinic-based enrollment provided participants with access to the digital maternity records and the option to select digital self-examination packages, including a standard care kit (weight scale, blood pressure cuff, urinalysis) or an innovative kit (smartwatch, sleep tracker). Among these, 110 (20.8%) did not select a package but chose to use the app, and 150 opted for the digital self-examination packages (28.4%). Of those, 2 (0.4%) chose the standard care kit only, 1 (0.2%) selected the innovative kit only, and 147 (27.8%) opted for both care packages (Fig. 1). At study start, 48 (13.3%) of the complete study cohort were pregnant in the first trimester, 191 (52.8%) in the second trimester and 123 (34.0%) in the third trimester. Of those who selected the self-examination packages, 2 (1.5%) were enrolled during the third trimester, 111 (84.7%) in the second and 18 (13.7%) in the first trimester (Table 1).

**Table 1:** Distribution of pregnancy week and trimester at study start for all participants and for the subgroup of participants that selected a smart self-examination package

Characteristic	Value	
	All participants (*n* = 528)	Participants with smart self-examination package (*n* = 150)
Pregnancy week, *n* (%)		
0–9	31 (8.6)	10 (7.6)
10–13	55 (15.2)	33 (25.2)
14–17	28 (7.7)	13 (9.9)
18–21	44 (12.2)	30 (22.9)
22–25	67 (18.5)	43 (32.8)
26–29	32 (8.8)	1 (0.8)
30–33	35 (9.7)	1 (0.8)
34–37	56 (15.5)	0 (0)
>38	14 (3.9)	0 (0)
*Not provided*	*166*	*19*
Trimester, *n* (%)		
First (0–11 weeks)	48 (13.3)	18 (13.7)
Second (12–27 weeks)	191 (52.8)	111(84.7)
Third (28–40 weeks)	123 (34.0)	2 (1.5)
*Not provided*	*166*	*19*

Absolute values are shown with relative proportions in parentheses. The number of participants with missing data is reported separately as *n* = [not provided] where applicable.

Demographic characteristics were submitted by 237 participants via the study app (Table 2). They had a median age of 33 years (IQR 30–36). Most participants were born in Germany (*n* = 214, 90.3%) and identified as German nationals (*n* = 213, 89.9%). Most participants had a partner (*n* = 232, 97.9%) and lived in households with 1–2 members (*n* = 132, 55.7%), with 56.5% (*n* = 134) having no and 34.6% (*n* = 82) having one child. Regarding education, 68.4% (*n* = 162) had obtained a high-school diploma (Abitur), and 59.1% (*n* = 140) held a college degree, while 8.9% (*n* = 21) had completed a PhD. Most participants are employed full-time during their pregnancy (*n* = 126, 53.2%), with 40.1% (*n* = 95) having reported a change in employment with their pregnancy. Additionally, 48.5% (*n* = 115) were on maternity leave at the time of the study. Technology use and interest were also assessed. Over half of the participants (*n* = 129, 54.4%) found technology “easy” to use, and 46.8% (*n* = 111) expressed a neutral interest in technology, while 16.9% (*n* = 40) were very interested. Sociodemographic characteristics of the subgroup of participants that opted for smart self-examination packages can also be found in Table 2. A similar distribution was observed for the subgroup who chose self-examination packages. However, in the self-examination group, the proportion of participants who were employed full-time during pregnancy was higher (*n* = 73, 60.3%), as was the proportion of participants with a PhD degree (*n* = 15, 12.4%).

**Table 2:** Sociodemographic characteristics of participants according to the sociodemographic questionnaire

Characteristic	Value	
	All participants (*n* = 528)	Participants with smart self-examination package (*n* = 150)
Country of birth, *n* (%)		
Germany	214 (90.3)	113 (93.4)
Other	23 (9.7)	8 (6.6)
*Not provided*	*291*	*29*
Nationality, *n* (%)		
German	213 (89.9)	111 (91.7)
Other	14 (5.9)	6 (5.0)
Multiple	10 (4.2)	4 (3.3)
*Not provided*	*291*	*29*
Partner, *n* (%)		
Yes	232 (97.9)	121 (100.0)
No	5 (2.1)	0 (0)
*Not provided*	*291*	*29*
Marital status, *n* (%)		
Married	167 (70.5)	88 (72.7)
Single	64 (27.0)	30 (24.8)
Divorced	5 (2.1)	2 (1.7)
Separated	1 (0.4)	1 (0.8)
*Not provided*	*291*	*29*
Household size, *n* (%)		
1–2	132 (55.7)	70 (57.9)
3–4	91 (38.4)	43 (35.5)
>4	13 (5.5)	7 (5.8)
Other	1 (0.4)	1 (0.8)
*Not provided*	*291*	*29*
Children in household, *n* (%)		
0	134 (56.5)	72 (59.5)
1	82 (34.6)	38 (31.4)
2 or more	21 (8.9)	11 (9.1)
*Not provided*	*291*	*29*
Highest school education, *n* (%)		
Still in school	1 (0.4)	0 (0)
Certificate of Secondary Education (Haupt- oder Volksschulabschluss)	12 (5.0)	6 (5.0)
General Certificate of Secondary Education (Mittlere Reife)	59 (24.9)	32 (26.5)
High-school diploma (Abitur)	162 (68.4)	83 (68.6)
No degree	3 (1.3)	0 (0)
Not provided	291	29
Highest degree of post-school education, n (%)		
No vocational training	9 (3.8)	4 (3.3)
In vocational training	7 (3.0)	3 (2.5)
Dual curriculum	60 (25.3)	29 (24.0)
College degree	140 (59.1)	70 (57.9)
PhD	21 (8.9)	15 (12.4)
*Not provided*	*291*	*29*
Change in employment during pregnancy, *n* (%)		
Yes	95 (40.1)	46 (38.0)
No	42 (59.9)	75 (62.0)
*Not provided*	*291*	*29*
Employment currently, *n* (%)		
Full time	126 (53.2)	73 (60.3)
Part time	59 (24.9)	25 (20.7)
Mini job	4 (1.7)	1 (0.8)
No employment	48 (20.3)	22 (18.2)
*Not provided*	*291*	*29*
Employment before, if it changed during pregnancy, *n* (%)		
Full time	56 (23.6)	31 (25.6)
Part time	27 (11.4)	8 (6.6)
Mini job	11 (4.6)	1 (0.8)
No employment	2 (0.8)	6 (5.0)
Not specified	1 (0.4)	0 (0)
*Not provided*	*291*	*29*
Maternity leave currently, *n* (%)		
Yes	115 (48.5)	41 (33.9)
No	122 (51.5)	80 (66.1)
*Not provided*	*291*	*29*
Subjective ease of technology use, *n* (%)		
Very easy	65 (27.4)	26 (21.5)
Easy	129 (54.4)	68 (56.2)
Neutral	36 (15.2)	23 (19.0)
Hard	6 (2.5)	4 (3.3)
Very hard	1 (0.4)	0 (0)
*Not provided*	*291*	*29*
Interest in technology, *n* (%)		
Very interested	40 (16.9)	19 (15.7)
Interested	63 (26.6)	35 (28.9)
Neutral	111 (46.8)	58 (47.9)
Not interested	16 (6.8)	7 (5.8)
Not interested at all	7 (3.0)	2 (15.7)
*Not provided*	*291*	*29*

Characteristics are reported as the number and percentage of the participants who submitted the respective information. Data are shown for the total study cohort (*n* = 528) and the subgroup, that selected smart self-examination packages (*n* = 150). For each characteristic, the number of participants with missing data is reported separately as *n* = [not provided] where applicable.

**Fig. 2:**
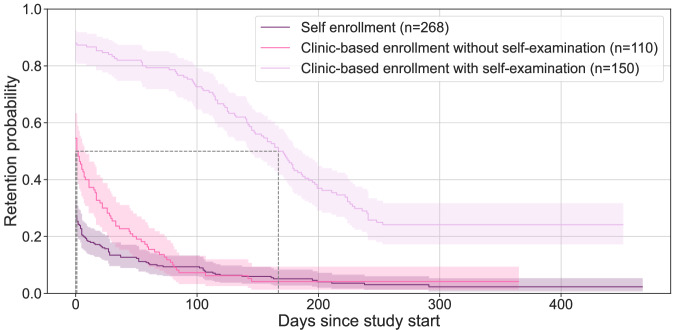
Kaplan-Meier survival curves showing retention probability over study period. Survival curves stratified by study cohort: Self enrollment, clinic-based enrollment without self-examination package, and clinic-based enrollment with self-examination package. Dashed lines indicate the median survival time (i.e., the point in time when 50% of participants have dropped out). The shaded region shows the 95% confidence limits. A log-rank test revealed statistical significance between study cohorts and intervention modalities (*p* < 0.005 for each comparison).

Survival analysis using Kaplan-Meier curves and a log-rank test revealed significant differences between the study cohorts (*p* < 0.005), Fig. 2. Self enrollment showed the shortest retention (median = 0 days, IQR = 0–2 days), followed by participants being enrolled via the clinics without self-examination package (median = 0 days, IQR = 0–33 days). The highest retention was observed among participants with self-examination package (median = 167 days, IQR = 96–248 days).

### App analytics

**Fig. 3:**
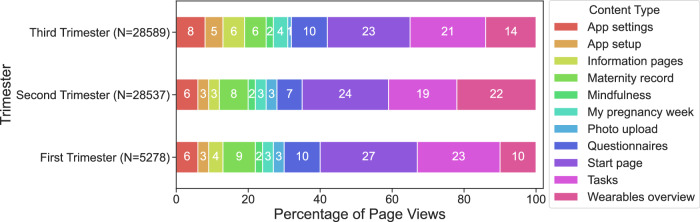
Percentual distribution of page views by content type across trimesters for the SMART start app. Values represent the proportion of total page views per content type within each trimester, given in %. Absolute numbers of page views are reported per trimester.

In total, participants had 64,675 interactions in the SMART Start app leading to 62,404 page views across 5,984 app sessions. The median duration of a user session was 61.0 s (IQR 13–337). Figure 3 shows the percentual distribution of page views by content type across the trimesters. The Start page accounted for the highest proportion of page views across all trimesters, with 27% in the first trimester, decreasing to 24% in the second and 23% in the third trimesters. Tasks were the second most interacted-with page, contributing 23, 19, and 21% of page views in the first, second, and third trimesters, respectively. Page views for the questionnaires section also showed minimal variation across trimesters, contributing 10% in the first trimester, 7% in the second and 10% in the third trimester. A detailed overview of page views by content is provided in Supplementary Table 1. We analyzed differences in app usage across trimesters among participants that had app activity in all three trimesters (*n* = 34). A two-way repeated-measures ANOVA revealed a significant main effect of Trimester, F(2, 66) = 9.21, *p* = 0.0004, η^2^_G_ = 0.042, indicating that total page view behavior changed over the course of pregnancy. A strong main effect of content type was observed, F(13, 429) = 66.20, *p* < 0.0001, η^2^_G_ = 0.392, suggesting that some content categories were consistently more frequently used than others. There was a significant interaction between Trimester and content type, F(26, 858) = 8.45, *p* < 0.0001, η^2^_G_ = 0.095, indicating that the pattern of content usage changed across trimesters, i.e., certain types of app content were accessed differently depending on gestational stage. Detailed results of ANOVA and the post hoc pairwise comparisons for each content type are provided in Supplementary Tables 2 and 3.

### Questionnaire participation and adherence to questionnaire schedule

**Fig. 4:**
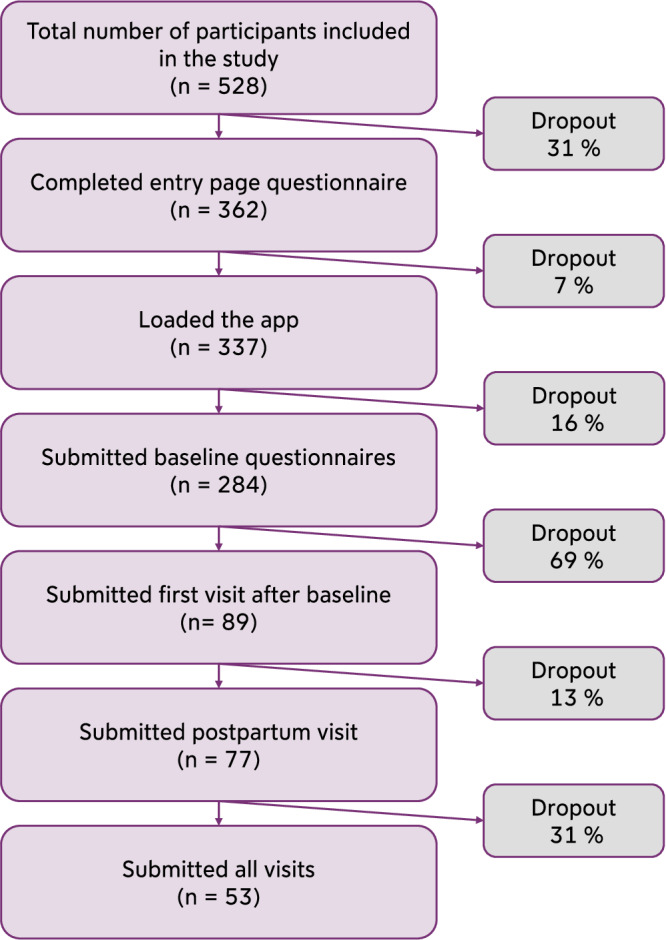
Dropout rates at different phases related to scheduled questionnaire visits of the study. The absolute numbers show the number of participants having reached a specific study phase. The dropout rates between two consecutive stages are given in percent.

Participant adherence related to scheduled digital questionnaire visits is summarized in a flowchart, illustrating dropout rates across specific study phases (Fig. 4). Among 528 participants initially included, 31% dropped out without completing the entry page questionnaire. Of those who completed the entry page questionnaire (*n* = 362), 7% did not proceed to load the app, and 16% of app users (*n* = 53) did not submit the baseline questionnaire. After completing the baseline questionnaire (*n* = 284), 69% of participants dropped out before completing the first visit after baseline. Following the first visit, 31% of participants dropped out before the postpartum visit, and ultimately, 53 participants (10% of the total) completed all intended visits.

Table 3 gives an overview of the total number of submissions for each questionnaire, while also giving also the frequency of participants having submitted 1, 2, 3, 4, 5 or more times a specific questionnaire. Among all questionnaires, the Cyberchondria Severity Scale (CSS) and the Pittsburgh Sleep Quality Index (PSQI) were scheduled for five submissions over the maximum study duration. Of those, the CSS has been submitted most often with 515 submissions and 16 participants having submitted five or more times. Multivariable Apnea Prediction Index (MAPI) questionnaire was scheduled for the time point ‘birth’ only and submitted accordingly by 78 participants. For the CSS and the health economic questionnaires some participants submitted more questionnaires than ideally intended by the study’s questionnaire schedule. An overview of the participant distribution over study visits is shown in Supplementary Fig. 1.

**Table 3:** Overview of participant submissions across various questionnaires, showing the frequency of submissions (from 1 to 5 or more times) and the total number of submissions for each questionnaire

	Number of participants having submitted a questionnaire …						Total submissions	Scheduled submissions	Number of participants total
	1×	2×	3×	4×	5×	more			
Edinburgh Postnatal Depression Scale (EPDS)	47	33	25	0	0	0	185	3	104
Multivariable Apnea Prediction Index (MAPI)	78	0	0	0	0	0	78	1	78
Pregnancy-related Health Behavior Scale (PHBS)	163	77	0	0	0	0	317	2	240
Pregnancy Physical Activity Questionnaire (PPAQ)	165	59	33	12	0	0	430	4	269
Pittsburgh Sleep Quality Index (PSQI)	135	40	44	26	1	0	456	5	246
Cyberchondria Severity Scale (CSS)	107	47	34	32	13	3	515	5	236
Sociodemographics questionnaire	237	0	0	0	0	0	237	1	237
Health economy questionnaire	148	73	1	0	0	0	297	2	222

The scheduled submissions are based on the SMART Start visit schedule of participants being included before week 12 of gestation.

Table 4 shows the view-to-completion rate and the scheduled completion rate for each questionnaire. The view-to-completion rate indicates the proportion of questionnaire views that resulted in an actual submission, reflecting participant engagement and the usability or perceived relevance of the questionnaire. The analysis shows variability across the questionnaires. For short and straight forward to answer questionnaires like the MAPI or the sociodemographic questionnaire, both the total view-to-completion rate and the median participant rate were high (MAPI: 84.8%, median 100.0%, IQR 100.0–100.0; sociodemographic: 82.3%, median 100.0%, IQR 100.0–100.0). In contrast, longer or more complex questionnaires (e.g. Pregnancy Physical Activity Questionnaire (PPAQ), PSQI or health economics questionnaires) exhibited greater inter-participant variability and lower view-to-completion rates (Table 4).

**Table 4:** View-to-completion rate (submitted questionnaires / questionnaire page views) and scheduled completion rate (submitted questionnaires / scheduled submissions), both reported in %

	Total view-to-completion rate in %	View-to-completion rate per participant, median (IQR) in %	Total scheduled completion rate in %	Scheduled completion rate per participant, median (IQR) in %	Number of questionnaire items
Edinburgh Postnatal Depression Scale (EPDS)	60.1	66.7 (50.0–100.0)	17.7	50.0 (20.0–75.0)	10
Multivariable Apnea Prediction Index (MAPI)	84.8	100.0 (100.0–100.0)	21.5	0.0 (0.0–100.0)	3
Pregnancy-related Health Behavior Scale (PHBS)	81.3	100.0 (66.7–100.0)	50.2	50.0 (50.0–100.0)	20
Pregnancy Physical Activity Questionnaire (PPAQ)	66.7	100.0 (50.0–100.0)	45.5	66.7 (33.3–100.0)	35
Pittsburgh Sleep Quality Index (PSQI)	64.7	80.0 (50.0–100.0)	41.6	50.0 (25.0–100.0)	26
Cyberchondria Severity Scale (CSS)	72.1	100.0 (60.0–100.0)	39.4	50.0 (20.0–75.0)	15
Sociodemographics questionnaire	82.3	100.0 (100.0–100.0)	65.5	100.0 (100.0–100.0)	18
Health economy questionnaire	34.8	30.0 (25.0–50.0)	43.6	50.0 (50.0–100.0)	11 / 31

The rate per participant is reported as median and interquartile range (IQR). The total number of items in each questionnaire is also shown. The health economy questionnaire was separated into two parts.

The scheduled completion rate reflects the proportion of submitted questionnaires relative to the scheduled submissions according to the protocol. It gives an estimate on how closely participants followed the intended study schedule for the respective questionnaires.

Scheduled completion rates also varied across questionnaires (Table 4). The highest total scheduled completion rates were observed for the sociodemographic questionnaire and the Pregnancy-related Health Behavior Scale (PHBS) with 65.5 and 50.2% respectively, both scheduled at the baseline visit. Lower rates were found for Edinburgh Postnatal Depression Scale (EPDS) and MAPI with 17.7 and 21.5%, respectively; both scheduled for the birth visit. Median participant-level scheduled completion rates ranged from 0.0% (MAPI) to 100.0% (sociodemographic questionnaire).

### Photo uploads

A photo upload was scheduled for the study participation for either to document urine tests or to digitally transfer information from their paper-based maternity record. A total of 3935 photos were uploaded by participants, consisting of 1291 urinalysis-related photos submitted by 101 participants and 2644 photos of various pages from 149 analog maternity records.

### Participation in self-examination and adherence to measurement guidelines

Participants were requested to perform home-based measurements according to a schedule. The highest number of datapoints for smart self-examination technology usage was collected for sleep episodes (30,084 datapoints from 108 participants, median 247, IQR 144–394, Table 5). The median sleep duration was 8 h and 6 min (IQR 5 h 6 min–9 h 27 min). Also, workouts (16,401 data points from 104 participants, median 105, IQR 36–215) and activity days were shared with a high number of participation (11,767 data points from 111 participants, median 103, IQR 43–137, Table 5). We examined whether the amount of data shared differed across participant subgroups defined by subjective ease of technology use, interest in technology, presence of children in the household, and highest school education. Mann-Whitney U tests for each subgroup–measurement combination revealed no significant differences. Detailed results are provided in Supplementary Table 4.

**Table 5:** Summary of data collected from smart self-examinations technology usage along with weekly protocol adherence rate to self-examinations

	Total number of datapoints	Number of participants	Average datapoints per participant, median (IQR)	Total weekly protocol adherence rate %	Weekly protocol adherence rate per participant, median (IQR) in %	Scheduled weekly measurements
Blood pressure	4,049	116	20 (8–48)	12.4	2.7 (0.0–11.5)	
Weight	4,377	128	22 (6–41)	42.0	44.8 (6.4–63.4)	
Sleep episodes	30,084	108	247 (144–394)	24.7	16.0 (0.0–45.2)	
Urinalysis	433	95	4 (1–7)	14.7	6.9 (0.0–18.6)	
ECG measurements	4,151	114	20 (6–39)	-	-	Not specified
Workouts	16,401	104	105 (36–215)	-	-	Not specified
Activity days (step count, elevation)	11,767	111	103 (43–137)	-	-	Not specified

The rate is defined as (weeks with full adherence to measurement protocol / total study weeks) and is reported in %. The rate per participant is reported as median and interquartile range (IQR). Data are presented as the total number of data points, the number of participants sharing each data type and the median (IQR) data points per participant. Workouts are defined as physical exercises lasting 10 min and longer. An activity day represents any day with recorded physical activity, such as steps taken or elevation changes, capturing general movement throughout the day. In total *n* = 150 participants opted in for the smart self-examination packages.

The adherence to the measurement schedule varied across modalities. Blood pressure was recorded in 12.4% of the weeks according to the predefined measurement schedule, while weight monitoring showed the highest adherence, with 42.0% of weeks completed as scheduled, followed by sleep episodes (24.7%) and urinalysis (14.7%). Participant-wise adherence rates are provided in Table 5.

**Fig. 5:**
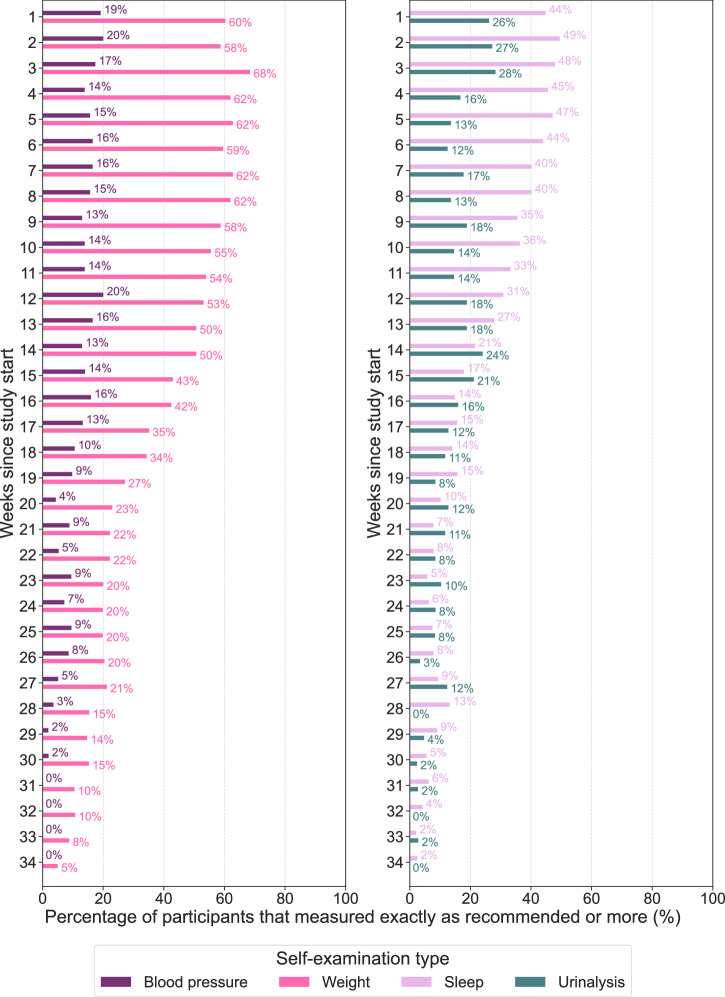
Adherence patterns for urinalysis, sleep, weight, and blood pressure over 34 weeks of study participation. The table displays the percentage of participants adhering exactly as recommended or more to the schedule for self-examination for each week. The percentage of participants adhering to the measurement recommendations are displayed on top of the bars. Percentages are based on participants who performed at least one measurement and whose individual study end date had not yet passed. A study end is defined as reaching 8 weeks after delivery or 8 weeks after week 40 of gestation, depending on the availability of the date of delivery.

We analyzed the weekly adherence to the measurement schedule over the course of study participation (Fig. 5). For urinalysis, adherence fluctuated considerably throughout the study period, with a peak adherence of 28% observed in week 3. This pattern indicates inconsistent engagement with urinalysis measurements over time. Sleep adherence showed a gradual decline after week 2, where a maximum of 49% of participants adhered exactly as recommended or more.

Adherence to weight measurements remained relatively stable, ranging from 50 to 68% up until week 14. This consistency suggests that participants were more likely to adhere to weight measurements than other types. Blood pressure adherence also exhibited stability, with a maximum adherence of 20% occurring in weeks 2 and 12. Despite this stability, the overall adherence levels for blood pressure measurements were lower than those for weight and sleep measurements.

Overall, adherence in the first weeks after study initiation varied between modalities. Weight monitoring showed the highest adherence probabilities at around 60%, followed by sleep monitoring at around 45%, and urinalysis at approximately 20%. Blood pressure measurements have the lowest adherence, with less than 20% of participants meeting the recommended guidelines.

Further, a notable drop in adherence was observed across all measurement types at week 14, potentially reflecting participant dropout or reduced engagement with the study. This trend is also visible in the survival curves of study participation (Fig. 2).

### Adherence patterns across participant subgroups

**Fig. 6:**
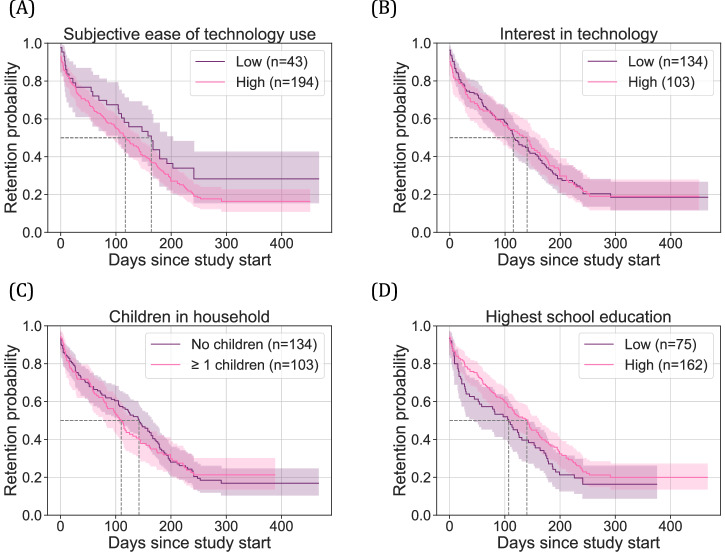
Kaplan-Meier survival curves showing retention probability over study period. Survival curves stratified by four self-reported participant characteristics: (**A**) Subjective ease of technology use, (**B**) interest in technology, (**C**) presence of children in the household, and (**D**) highest level of education. Dashed lines indicate the median survival time (i.e., the point in time when 50% of participants have dropped out). The shaded region shows the 95% confidence limits. Log-rank tests did not reveal statistically significant differences in retention among the groups.

We analyzed the retention probability of participants among different self-reported subgroups, including perceived ease of technology use, interest in technology, presence of children in the household, and highest level of education. Survival curve analysis revealed no statistically significant differences in the duration of participation until dropout across these groups. All subgroups showed comparable retention trajectories over time (Fig. 6).

Table 6 shows the weekly protocol adherence rates for self-examination stratified by participant characteristics. We performed group comparisons using Mann-Whitney U tests. Significant differences in adherence were observed across several subgroups. Participants who reported higher subjective ease of technology use demonstrated greater adherence to weight measurements compared to those with lower ease of use (50.0% vs. 22.9%, *U* = 885.5, *p* = 0.017, *r* = 0.302). Similarly, participants with greater interest in technology showed significantly higher adherence rates for both blood pressure (5.0% vs. 0.0%, *U* = 1418.0, *p* = 0.033, *r* = 0.216) and weight measurements (52.1% vs. 38.9%, *U* = 1378.5, *p* = 0.025, *r* = 0.238). The presence of children in the household was associated with lower adherence: participants without children had significantly higher adherence for blood pressure (3.8% vs. 0.0%, *U* = 2169.5, *p* = 0.025, −0.230), weight (51.0% vs. 20.8%, *U* = 2328.0, *p* = 0.003, *r* = −0.320), and urinalysis (12.9% vs. 4.0%, *U* = 2156.0, *p* = 0.038, *r* = −0.222). Finally, participants with a higher educational showed significantly higher adherence for weight (50.0% vs. 22.9%, *U* = 1074.5, *p* = 0.005, r = 0.319) and urinalysis measurements (12.0% vs. 3.9%, *U* = 1160.5, *p* = 0.019, *r* = 0.264).

**Table 6:** Weekly protocol adherence rate (weeks with full adherence to measurement protocol / total study weeks) for four self-examination modalities and scheduled completion rate for all questionnaires (submitted questionnaires / scheduled submissions) across four self-reported participant characteristics: subjective ease of technology use, interest in technology, presence of children in the household, and highest school education

	Scheduled completion rate per participant, median (IQR) in %	Weekly protocol adherence rate per participant, median (IQR) in %			
Characteristic	All questionnaires	Blood pressure	Weight	Sleep	Urinalysis
Subjective ease of technology use					
Low (*n* = 43)	46.2 (31.6–84.2)	2.4 (0.0–6.3)	22.9 (4.1–51.1)*	7.7 (0.0–38.8)	4.0 (0.0–12.7)
High (*n* = 194)	52.9 (35.3–84.2)	3.8 (0.0–14.2)	50.0 (17.7–66.7)*	21.5 (3.9–50.0)	11.9 (2.9–19.9)
*p-value*	*p* = *0.408*	*p* = *0.315*	*p* = *0.017*	*p* = *0.157*	*p* = *0.065*
Interest in technology					
Low (*n* = 134)	47.4 (31.6–84.2)	0.0 (0.0–10.1)*	38.9 (4.3–62.2)*	11.1 (0.0–46.2)	6.5 (0.0–19.7)
High (*n* = 103)	56.2 (35.3–79.5)	5.0 (0.0–14.2)*	52.1 (33.0–66.7)*	24.4 (7.9–49.5)	11.7 (2.9–18.6)
*p-value*	*p* = *0.545*	*p* = *0.033*	*p* = *0.024*	*p* = *0.063*	*p* = *0.352*
Children in household					
No children (*n* = 134)	62.0 (31.6–89.2)	3.8 (0.0–16.7)*	51.0 (31.8–67.0)**	27.0 (6.7–50.0)	12.9 (3.6–22.9)*
≥1 children (*n* = 103)	46.2 (35.1–78.2)	0.0 (0.0–6.7)*	20.8 (4.0–55.6)**	8.0 (0.0–33.3)	4.0 (0.0–16.7)*
*p-value*	*p* = *0.107*	*p* = *0.025*	*p* = *0.003*	*p* = *0.056*	*p* = *0.038*
Highest school education					
Low (*n* = 75)	46.2 (35.3–77.9)	0.0 (0.0–7.6)	22.9 (5.2–57.1)**	15.4 (0.0–37.1)	3.9 (0.0–14.6)*
High (*n* = 162)	56.4 (31.6–84.2)	3.8 (0.0–15.1)	50.0 (24.5–69.3)**	22.2 (3.3–50.8)	12.0 (3.6–19.7)*
*p-value*	*p* = *0.256*	*p* = *0.197*	*p* = *0.005*	*p* = *0.253*	*p* = *0.019*

Rates are reported as median and interquartile range (IQR) in %. Group comparisons were conducted using Mann-Whitney U tests. *P*-values for all comparisons are reported in the table. Statistical significance is indicated as *p ≤ 0.05, **p ≤ 0.01, ***p ≤ 0.001.

No statistical significance was found for scheduled completion rates of questionnaires among the subgroups, indicating that the questionnaires were completed according to the schedule at similar rates across all participant subgroups, regardless of technological affinity, household composition, or educational background. Group differences among the individual questionnaires are shown in Supplementary Table 5.

## Discussion

This work presents a comprehensive digital concept for smart pregnancy care. We developed an all-in-one solution that used a mobile health app as a foundation, which could be connected with smart sensor devices. Further, the app included a digital maternity record and comprehensive pregnancy-supportive content for educational purposes as well as self-reflection tools. This study adds to the growing body of research on digital maternal health by implementing and evaluating an integrated, app-based prenatal care model in a real-world, high-income setting. Based on the adherence-related results, we demonstrate the general feasibility of an at-home study with digital tools in pregnancy care. With this concept, we established an infrastructure for large-scale continuous data collection of self-reported and sensor-derived health data. In this work, we did not analyze wearable data in detail. Rather, we provided an overview of the data that can be collected using a digital framework for pregnancy care and the difficulties observed within such a digital study concept.

### Consistent adherence to self-examination demonstrates the feasibility of integrating routine prenatal care interventions in home environment

Our study provides initial evidence that the integration of self-monitoring examinations from routine pregnancy care into a home-based setting is generally feasible. However, adherence levels highlight relevant limitations for real-world implementation. Moderate engagement with weight monitoring indicates practical barriers or motivational issues that may affect participants' compliance. Lower adherence to blood pressure monitoring may be attributable to a more demanding measurement protocol. Nevertheless, the stable adherence trend over multiple weeks suggests feasibility for sustained participation if protocols are manageable. Additionally, the substantial engagement of participants who performed urinalysis at home, indicates intrinsically motivated engagement. Compared to other mHealth studies of similar duration, adherence in our study demonstrated sustained engagement with self-monitoring. In the Datamama study by Gerbier et al., questionnaire participation reached 73%, but long-term adherence data were not available^27^. Bush et al. reported that only 41% of users remained active month-to-month over a 6-month period^28^. Our findings underscore the practicality and the willingness of pregnant individuals to engage in home-based testing. Future research should evaluate the accuracy of urinalysis, blood pressure and weight self-examinations that have been captured within this longitudinal at-home study environment compared to standard prenatal care to assess their clinical utility and reliability.

The SMART start study investigated the acceptance and technical feasibility of home-based pregnancy care without changing anything from the routine antenatal care. This must be taken into account when evaluating the adherence to the scheduled measurements. In future studies, it would be worth exploring whether replacing parts of the standard care with home-based monitoring improves adherence. The knowledge that such self-examinations are a crucial part of pregnancy care—rather than being just an add-on—could help to shift the responsibility and increase motivation and overall compliance.

### Adherence to digital questionnaires highlights challenges and design priorities for longitudinal app-based monitoring

Our findings highlight the potential and limitations of integrating digital questionnaires into longitudinal pregnancy monitoring. While engagement upon accessing questionnaires was generally high, overall adherence to scheduled questionnaire submissions was substantially lower. Adherence was highest for short, low-burden questionnaires, while more demanding or sensitive instruments showed reduced completion. These results underline the importance of timing, content design, and perceived relevance when integrating questionnaires into digital health tools. Future digital pregnancy interventions should consider adaptive, context-aware scheduling and optimized user experience to maintain adherence and ensure high-quality longitudinal data collection.

### Integrating novel wearable-based measurements beyond standard prenatal care creates opportunities to uncover new biomarker-outcome relationships

A strength of our study lies in the generation of a rich, multimodal dataset that combines traditional clinical measurements with continuously collected digital biomarkers. This unique data infrastructure enables us to explore novel associations between behavioral and physiological patterns during pregnancy and clinically relevant outcomes. By linking digital biomarkers, such as activity, sleep, or heart rate variability, with both classical biomarkers and pregnancy outcomes, we can gain new insights into maternal and fetal health trajectories. Beyond the conventional measurement modalities of routine prenatal care, our study incorporated novel wearable-based assessments, including a mattress for sleep analysis and a smartwatch. Recent research shows that sleep quality and physical activity during pregnancy influence the risk of preterm birth and other pregnancy-related complications. A meta-analysis by Wang et al. indicated a potential link between poor sleep quality or short sleep duration and an increased risk of preterm birth^29^. Similarly, a systematic review by Schlüssel et al. identified associations between regular physical activity during pregnancy and favorable outcomes, such as reduced risk for excessive gestational weight gain, gestational diabetes, and adverse birth outcomes^30^. However, evidence remains limited due to the scarcity of high-quality data. Our framework enables continuous and unobtrusive collection of sleep and activity data throughout pregnancy, addressing this critical gap.

### Enabling large-scale longitudinal data collection in home-based pregnancy care

The SMART Start framework represents a significant advancement in digital pregnancy monitoring by establishing a scalable infrastructure for large-scale, longitudinal data collection with high granularity. This was made possible through an all-in-one digital implementation, where a smartphone app serves as the central hub for data acquisition, integration, and synchronization. The system seamlessly combines multiple data sources, including automatically synchronized sensor data, integrated digital questionnaires, and an embedded digital maternity record, enabling continuous data collection with minimal participant burden.

To the best of our knowledge, our study provides the most comprehensive dataset in terms of complexity and integration of digital health technologies for home-based pregnancy care. We included 528 pregnant individuals who participated on average for 25 weeks. Compared to previous digital pregnancy studies, our dataset exceeds such approaches in scope or diversity of data. While previous studies may have included a higher number of participants, they typically focused on a single data source, such as wearable-derived physiological signals^22,31^, questionnaire-based insights or app analytics^27^. Few studies combine data sources^32,33^.

Sugawara et al.^33^ focused on self-reported lifestyle data and physiological measurements from home healthcare devices but lacked user-centric engagement features that could sustain long-term adherence. While they successfully integrated multiomics data, their dataset was limited in breadth regarding behavioral and engagement-related aspects. Similarly, Gerbier et al.^27^ relied on questionnaire-based data collection via a pregnancy app. However, they did not integrate wearable devices or app analytics. Other digital pregnancy care concepts, such as OB Nest^34^ and SAFE@HOME^35^ incorporated remote monitoring and clinical integration but did not offer a unified app-based system with behavioral analytics or were focused on a specific pregnancy-related disease. In contrast, SMART start combines self-reported information from questionnaires, objective wearable-derived physiological data, and app analytics that capture not only medical parameters but also behavioral trends and adherence patterns over time.

### Enhancing user engagement through comprehensive pregnancy-supportive content

User engagement is a critical determinant of adherence in digital health interventions. A review by Jakob et al. identified various factors that influence adherence positively, including personalization of mHealth content, reminders in the form of individualized push notifications, and a user-friendly and technically stable app design^36^. To incorporate personalized content, we provided pregnancy-related information tailored to the user’s gestational week, ensuring that content remains relevant and meaningful throughout pregnancy. To support adherence through reminders, we implemented individualized push notifications, reminding users of pending tasks, such as questionnaire completion. Finally, to ensure a seamless and user-friendly experience, the app was developed through an interdisciplinary process, integrating insights from healthcare professionals, computer scientists, and usability experts. A prior usability study with selected app contents was conducted to optimize functionality and technical stability before deployment^37^.

Further, literature suggests that factors that offer direct benefits to users can foster sustained engagement with digital health applications^38^. Therefore, we included features, such as educational content, a pregnancy journal and MBSR audio exercises. Our findings highlight the importance of designing digital health tools that not only collect data but also provide tangible value to users.

### Multidimensional adherence assessment reveals early dropout and varying engagement in self-guided mHealth study

While previous pregnancy-related studies on adherence in mHealth have mainly focused on specific features, such as their impact on outcomes or adherence to a single protocol^28,39,40^, there remains a gap in understanding how adherence manifests in large-scale, multifaceted digital health studies.

Adherence in mHealth studies is measured in several ways that strongly depend on the underlying study design^33^. In our analysis, we evaluated adherence across multiple dimensions using quantifiable metrics commonly applied in comparable mHealth studies, such as usage logs^41^, survey completion rates^42^, number and length of app sessions^43^ or interaction with wearables^12^.

However, directly comparing retention of dropout rates is challenging due to differences in study protocols and engagement criteria. In our multimodal study design, participants could remain active even if they discontinued specific study components, such as questionnaires, while continuing to share wearable data.

We observed a high dropout rate within the first weeks of the study. One possible explanation is the low barrier to entry due to self-registration, making it equally easy for participants to disengage shortly after enrolling. The dropout rate was higher among participants without a digital package, possibly because they had access to fewer app features. Key elements like the digital maternity record or self-examinations were only available with extended participation, which may have reduced engagement. Other mHealth studies also report high attrition in the initial days of remote digital health interventions^44,45^. We observed varying view-to-completion rates among the different questionnaires. Short and straightforward questionnaires were completed more often than long and complex questionnaires. These findings suggest that questionnaire length and complexity might influence participant engagement and completion rates^46,47^. We did not implement a follow-up mechanism, which limits the investigation of participant motivations for disengagement. Understanding these factors is important for improving future digital health interventions.

### Unequal engagement across user groups underscores the need for inclusive, context-aware digital health design in pregnancy

Our overall goal was to create a digital pregnancy care framework applicable to a broad population of pregnant individuals. Therefore, we deliberately included no restrictions regarding risk profile, comorbidities, or sociodemographic characteristics. However, we are aware that such technologies are not equally effective or accessible for all pregnant women. Particularly in high-risk pregnancies, remote monitoring can provide a sense of additional safety and reassurance for the expectant mother and represents a central use case for digital prenatal care. Prior studies have shown that home-based telemonitoring can be a safe alternative to inpatient or frequent outpatient care in high-risk pregnancies, while offering women the comfort of staying in their own environment during a vulnerable period^48,49^. In this subgroup of high-risk pregnancies, adherence to digital care protocols may be higher due to the direct perceived benefit of early detection and closer surveillance^50^. Socioeconomic, technological, and individual-level factors can further significantly influence engagement, usability, and perceived benefit. Unequal access to devices and digital literacy, especially among vulnerable populations, such as refugee women or those with mental health burdens, contributes to digital exclusion^51,52^. Age, education, and e-health literacy have also been shown to shape digital health behavior and risk of cyberchondria, underscoring the need for tailored content and appropriate onboarding strategies^53^. Consistent with this literature, our study revealed marked differences in adherence across user groups: participants with higher educational attainment and stronger interest in technology showed greater adherence to self-monitoring tasks, while those with existing children demonstrated significantly lower engagement across some interventions. Furthermore, participants who were onboarded in person at clinical sites remained active longer than those who enrolled independently online, emphasizing the importance of structured introduction and support. While adherence analyses revealed meaningful group-level differences in compliance with specific self-examination protocols, the overall retention time did not differ between participant subgroups. This discrepancy highlights that continued participation in the study does not necessarily equate to consistent engagement with all components of the digital care model. For example, participants with higher technical affinity or without children in the household did not remain in the study longer but were more likely to adhere to scheduled measurements, such as weight and blood pressure. This suggests that while general retention may be influenced by broader motivational or structural factors, adherence to specific tasks is more sensitive to individual characteristics, such as perceived usability, available time resources, or personal benefit from self-monitoring. These findings highlight the importance of differentiating between study retention and protocol adherence when evaluating engagement with digital health interventions. The representativeness of our findings may be limited, as participants were predominantly highly educated and showed strong interest in technology, potentially reducing generalizability to broader pregnant populations. Future studies should consider tailoring intervention intensity or user support based on individual user profiles, especially when adherence to more effortful tasks is needed. As others have argued, equity-focused and co-designed approaches are essential to ensure that digital maternal health tools serve the full spectrum of pregnant individuals^52,54^.

### Lessons learned

The SMART Start study offered valuable insight into the implementation of digital tools into routine pregnancy care including self-examination technologies and the transfer of routine measurements into a patient-sovereign home care setting. Several lessons emerged from the conducted trial that should guide future implementations.

Implementing feedback loops: One key issue was the lack of clinically relevant feedback from the wearables and urine measurements provided by the app. Participants could see changes in heart rate, sleep duration or blood pressure over time but did not receive any feedback on whether the data were normal or if action was needed. Without some form of data interpretation or clinical guidance, measurements can feel disconnected from care, and the engagement to collect such data could decrease over time. Similar patterns have been reported in other digital health contexts, where missing clinical relevance or actionable feedback can reduce sustained use and user motivation^55,56^. For future versions of such eHealth apps it would be beneficial to provide in-app feedback, e.g., using a traffic light system or a similar logic, to return quick and easy-to-understand information on the measurements’ results to the respective user. For such feedback loops, machine-learning-based approaches could further help to flag values based on the need for attention not only for the users but also for caregivers.

Providing feedback on data transmission: In a similar context, some participants gave feedback that they weren’t sure whether their data was successfully transferred to the study center and whether the measured values reached the treating physicians. A simple confirmation, e.g., checkmarks showing successful transmission, could improve the trust into the system and data transfer, potentially leading to a higher study schedule adherence.

Adapt self-examination schedule to individual risk levels: Engagement with passive measurements, such as activity tracking via smartwatch or sleep tracking using the smartwatch or sleep mat, was relatively high. In contrast, active measurements like manual blood pressure recordings or urine tests were completed less often than scheduled. In future implementations, a selective approach, in particular for manual, active measurements, with a high frequency of measurements as part of an intensive monitoring schedule only for e.g., high-risk pregnancies, while the measurement for low-risk cases could be kept only as frequent as necessary, defined by the standardized routine schedules. Adherence may also depend on risk perception. Women with high-risk pregnancies or increased pregnancy-related anxiety may be more motivated to stick to the defined schedule due to intrinsic motivation to monitor their own as well as the baby’s health^57^.

Better enrollment and continuous support: From a study logistics perspective, higher individual support during the enrollment phase, such as filling out the baseline questionnaires together or providing teaching for the key app features, might improve early retention. In particular, early disengagement was a key issue that needs to be addressed in future studies. Furthermore, support and engagement during the study phase could be strengthened by actively involving practicing gynecologists in the study's conduct. If digital tools and adherence to the defined monitoring schedule were routinely addressed during standard pregnancy care visits, it could reinforce the importance of participation and improve the overall compliance. Additionally, digital strategies to enhance onboarding and retention, such as personalized content based on questionnaires or wearable data, gamification elements, or companion apps, could further support sustained engagement throughout the study.

Fostering interdisciplinary understanding: SMART Start involves experts from five domains, each with distinct workflows and challenges. Introducing the specific realities of these fields can foster mutual understanding and improve interdisciplinary collaboration. Examples include clinical internships and good clinical practice training for technical professionals, as well as introductory sessions on agile development methodologies for medical staff.

Structured requirements engineering and agile development: In the SMART Start project, evolving technical and clinical needs highlighted the difficulty of fully specifying requirements at the outset. Digital health research projects are often characterized by limited initial predictability, with the feasibility of specific functionalities becoming clear only during project execution. Employing systematic requirements engineering, including their definition, prioritization, and iterative implementation with an agile development framework, allows for flexible adaptation to these inherent uncertainties.

Patients-first approach and iterative user experience evaluation: Digital health solutions must deliver tangible value to users while ensuring a positive overall user experience to facilitate adoption. This necessitates the systematic and continuous involvement of patients and end-users throughout the development process. Importantly, assumptions about user needs should be validated through empirical testing rather than based solely on expert opinion. Methods, such as prototyping, iterative user experience assessment, and structured run-in phases, support the development of user-centered and clinically relevant solutions.

### Future work and potential improvements

In order to establish our digital pregnancy care concept in practice, some points need to be discussed, regulated or developed in the future. A dedicated interface for caregivers is necessary to ensure that the collected data is practically usable in routine pregnancy care. The system needs clear protocols for managing outlier or critical health measurements. Further, defining when and how users receive alerts, guidance, or emergency contact recommendations is essential for ensuring safety and effective self-monitoring. Regarding the innovative sensor technology established in this study concept, further evidence is needed to demonstrate its direct impact on improving health outcomes or the overall quality of care. The high dropout we observed in early study participation highlights the need for a better onboarding and retention strategy. Additional studies need to investigate how adherence behaves when aspects of the standard care are replaced by home-based monitoring.

## Methods

### Study design

The SMART start study was designed as an open, prospective, monocentric, interventional feasibility study to investigate comprehensive aspects of a concept for the digital, preventive care of pregnant individuals. The study was conducted at the Department of Obstetrics and Obstetrics of the UKER. Participant recruitment started in January 2022 and ended in January 2024.

**Fig. 7:**
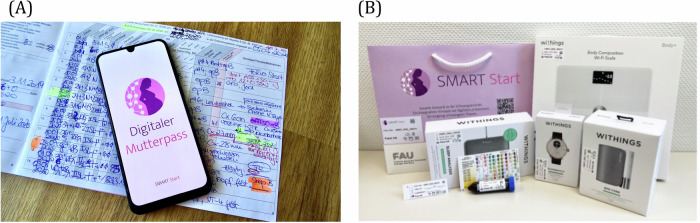
The SMART start concept integrates a mobile application with digital self-monitoring technologies for pregnancy care. **A** SMART Start app including maternity record function with **B** connected smart self-examination technologies.

**Fig. 8:**
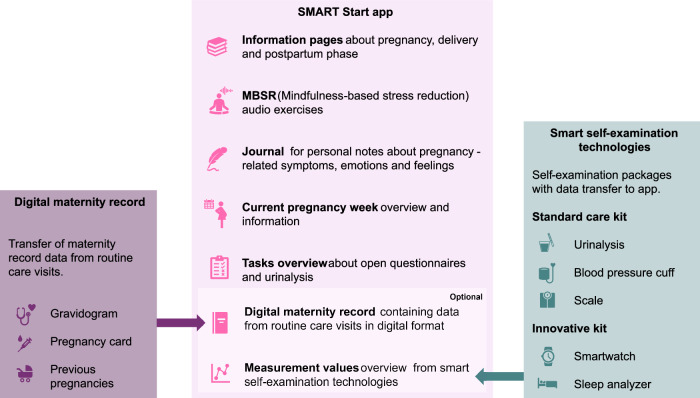
Overview of the components of the SMART Start study. The SMART Start app combines functionalities that enhance user engagement and provide informational content, like personalized information pages, a journal function and Mindfulness-based stress reduction (MBSR) exercises. Further, it gives an overview of weekly open tasks like open questionnaires or urinalysis. The smart self-examination technologies combine the standard care kit and the innovative kit. Respective measurement values are automatically transmitted to the SMART Start app. Important metrics from the routine care visits can be accessed in the digital maternity record including the gravidogram (chart used in prenatal care to monitor fetal growth and pregnancy progression), information about previous pregnancies and the pregnancy card containing relevant information in case of an emergency. Participants could opt in for the digital maternity record or / and for the smart self-examination packages.

The evaluated system consists of a smartphone application, the SMART Start app, that included a digital maternity record functionality and smart self-examination technologies, as shown in Fig. 7. The smart self-examination technologies comprised two packages: the standard care kit, which included components for simple, established measurements for prenatal care that can be easily integrated into the home environment, and the innovative kit, which contained advanced measurements that are not yet established in regular prenatal care. Figure 8 gives an overview of the components of the SMART start study, which are explained in detail in the following chapters: Smart Self-Examination Technologies, SMART Start App and Digital Maternity Record. The app and the study were designed in line with user-centered design and underwent usability evaluations and improvements beforehand. The interventions implemented in this study were designed to not impact or alter routine clinical care practices. Data collected through self-measurements were not routinely reviewed by healthcare professionals during the study. Participants were able to show their measurements to their treating physicians during standard prenatal care visits, based on their own decision, using the SMART Start app. This study was designed as a feasibility trial. Therefore, no formal power calculation was performed.

### Ethical approval, trial registration, and data protection

The SMART Start study was approved by the institutional review board of the Friedrich-Alexander-Universität Erlangen-Nürnberg (530_20B) and was registered in the German Clinical Trials Register under the identifier DRKS00036867. The study was carried out in compliance with the ethical principles set out in the Declaration of Helsinki and applicable local laws. To ensure compliance with data protection regulations, a comprehensive data protection and IT security concept was developed for this study. This concept was reviewed and approved by both the data protection officer and the IT security officer of the university hospital Erlangen. All technical and organizational measures follow the requirements of the General Data Protection Regulation (GDPR). Given the involvement of wearable devices from an external manufacturer (Withings), particular emphasis was placed on ensuring secure and GDPR-compliant processing of health-related data. Data transmission and storage were designed in accordance with European data protection standards. Participants provided informed consent for the use of pseudonymized health-related data. The SMART Start app operated on a secure, hospital-hosted server infrastructure with Hypertext Transfer Protocol Secure (HTTPS) -encrypted data transmission and strict access controls. All app interactions, including questionnaire responses, maternity record uploads, and urinalysis images, were stored on internal systems. File uploads (e.g., urinalysis) were routinely screened for malware. Participants who used wearable devices (Withings) had to create an account and accept Withings’ data protection policy in accordance with GDPR; data were stored on Withings’ European cloud servers and retrieved pseudonymously via token-secured application programming interfaces (APIs). The study server’s communication was restricted through a dedicated Virtual Local Area Network (VLAN) and firewall configuration, allowing only specific outbound/inbound traffic (e.g., secure API access, operating system updates). All data exchanges with internal clinical systems were managed via secure Open Database Connectivity (ODBC) and ActiveX Data Objects for .NET (ADO.NET) interfaces, and data from the app was periodically integrated into the hospital’s Microsoft Structured Query Language (MSSQL) system for analysis via structured Extract, Transform, Load (ETL) processes. The system was subject to routine monitoring and patching by the hospital’s IT security team. Study participants were informed about their rights under the GDPR, including the right to access, correct, or withdraw their data collected within the SMART Start study. Anonymous app use was also offered for participants without device packages, providing an additional privacy-preserving option.

### Eligibility, screening, and consent

Participants were informed and recruited via the UKER. In addition, a project website was launched for interested individuals across Germany. All potential study participants had to self-register to the study via the SMART Start app. They completed a questionnaire to screen for eligibility, and those meeting criteria received detailed study information. All participants provided informed consent before being enrolled in the study. The use of the SMART Start app was anonymous for those who have not been enrolled at the UKER. For those, participation took place via self-registration in the app, allowing pregnant individuals from all over Germany to take part in the study. Study participants recruited via the UKER provided a written informed consent for the use and transfer of pseudonymized data. Study participants used their private smartphones. The smart technologies for self-examinations were provided by the UKER. For participants who choose a digital package, study participation was pseudonymized. In contrast, participants without a device package could choose between using the app anonymously or pseudonymously throughout the study. A pseudonymous use of the app enabled the digitalization feature of their maternity record. Only the opt-in for a pseudonymous data transfer allowed the digitalization of the paper-based record.

### Inclusion and exclusion criteria

Participants were pregnant individuals aged 18–50 years who provided informed consent for study participation. Exclusion criteria for the self-examinations were mostly based in the instructions for use of the smart devices. These included a gestational age outside the range of 8 + 0 to 23 + 6 weeks, the presence of electrical implants as well as physical limitations, such as arm circumference (<22 or >42 cm) or body weight (>180 kg) for the standard care kit. For the innovative kit, exclusion criteria included mattress thickness (<10 or >40 cm), wrist circumference (>20 cm), and poor skin integrity at the wrist.

### Smart self-examination technologies

The smart self-examination technologies were structured in two packages. We focused on devices from the manufacturer Withings Ltd. (Issy-les-Moulineaux, France), referred to as Withings in the following. The standard care kit included components for simple, established measurements for prenatal care that can be easily integrated into the home environment. It consisted of a body composition scale for weight control (Withings Body + ), an upper arm blood pressure cuff (Withings BPM Core), and urine test strips (Multistix 10 SG Reagent Strips, Siemens Healthineers AG, Erlangen, Germany).

The innovative kit contained innovative measurements that are not yet an integral part of regular prenatal care. It included a smartwatch (Withings ScanWatch) and a sleeping mat (Withings Sleep Analyzer). These were intended to quantitatively capture sleep and activity parameters throughout the course of pregnancy.

A picture of the smart self-examination technologies is shown Fig. 7, an overview of the packages is given in Fig. 8.

### SMART start app

The SMART Start app served as the central component of the SMART Start study. It combined study functionality, pregnancy-related questionnaires, and pregnancy-supportive content, an interface to smart self-examination technologies, and a digital maternity record functionality.

An overview of the pages included in the SMART start app is shown in Fig. 8. Its functionalities were defined by an interdisciplinary team of gynecologists, computer scientists, ethicists and health economists, incorporating insights from a prior study^58^.

The SMART Start app was available only in German, as the study was conducted in Germany and the majority of participants were German-speaking. Screenshots of all app pages translated to English and the original German content can be found in the Supplementary Figs. 3–10.

**Fig. 9:**
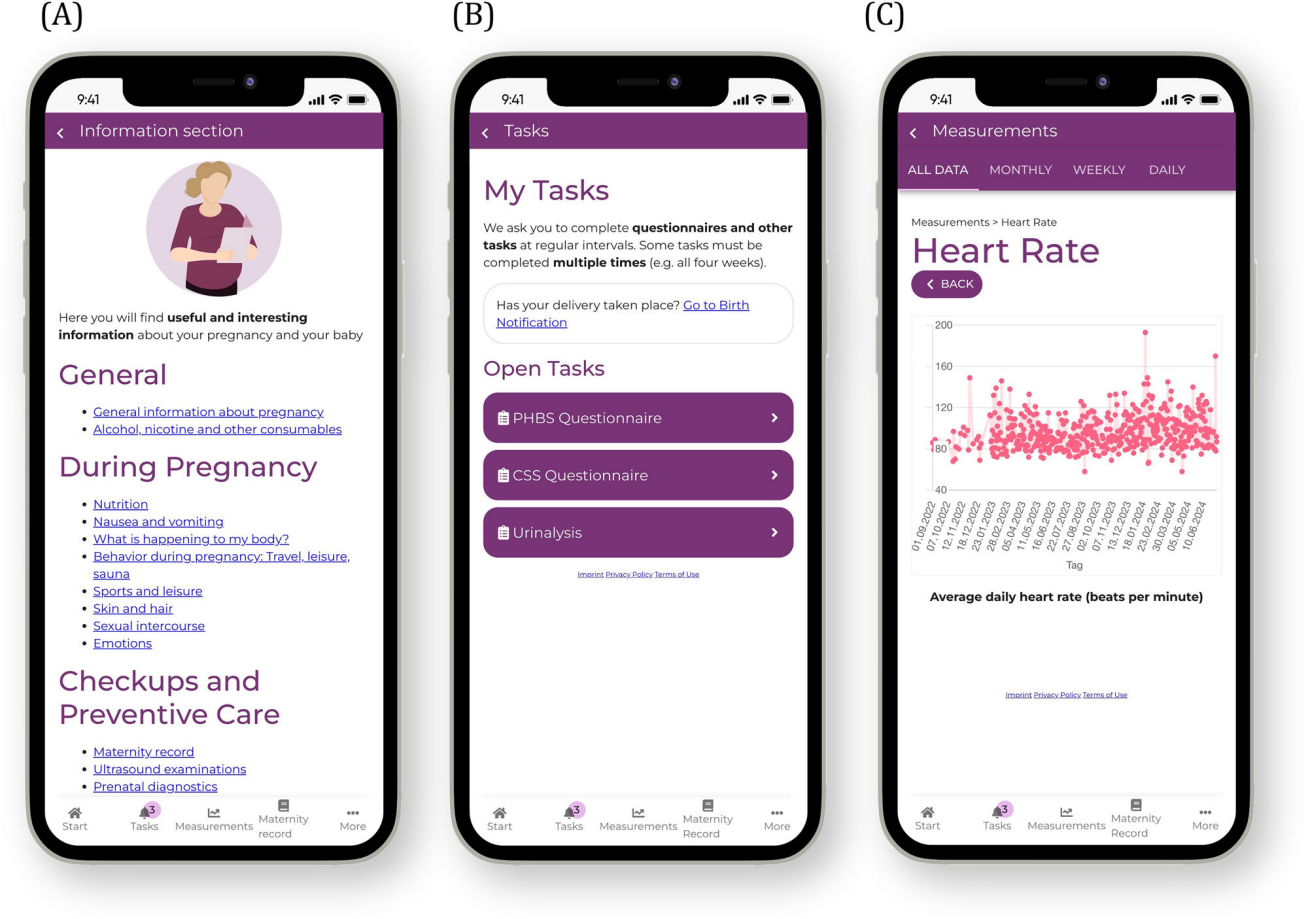
Screenshots of the SMART start app. **A** Information section containing pregnancy-related material and recommendations. **B** Tasks overview page providing a summary of weekly to-dos. **C** Self-examination values for heart rate in a weekly overview.

The app included features that were designed to enhance user engagement and provide informative content. We implemented information pages that contained general pregnancy-related information and recommendations (Fig. 9). All texts and recommendations have been carefully developed by physicians from the UKER. Further, we added a section for mindfulness-based stress reduction (MBSR) exercises. The recordings were narrated by the Department of Psychiatry and Psychotherapy of the UKER. A journal function gave the user the possibility to track their daily emotional state. In the app section, my pregnancy week, the user could find personalized information that was dependent on the current week of gestation. A dedicated tasks overview page summarized weekly to-dos, such as questionnaires and self-administered urinalysis (Fig. 9). For the self-administered urinalysis, the SMART start app included a step-by-step introduction, which was built on a previous study^59^.

The SMART Start app further built an interface for the smart self-examination technologies. Selected **measurement values** from the Withings devices were automatically transferred to the app where they were graphically displayed (Fig. 9). A flowchart illustrating the in-app workflow of a user journey is provided in Supplementary Fig. 2.

The SMART Start app was developed as a React-based progressive web app (PWA) with a Node.js/MySQL backend, hosted on secure servers at University Hospital Erlangen with firewall protection and vulnerability monitoring. Regular data transfer to the electronic case report form (eCRF) ensured data quality. Device data were stored on Withings’ cloud servers and accessed via Representational State Transfer Application Programming Interface (REST API), enabling periodic synchronization with the SMART Start server. Data transmission occurred continuously throughout the study.

### Digital maternity record

A central element of the SMART Start concept was the digital maternity record, which translates the contents of the traditional paper-based maternity record into a digital format. Essential information from the analog maternity record was systematically transferred into the digital version integrated into the SMART Start application continuously throughout the study period. Participants submitted photographs of their analog maternity records via the app, which were then reviewed and digitized by the study team at the UKER. The digitized data were subsequently made available to participants within the app. Specifically, three pages of the analog maternity record were digitized: the gravidogram, previous pregnancies, and a pregnancy card, which summarizes selected additional maternity record data. The gravidogram is a chart that is used during prenatal care to monitor fetal growth and pregnancy progression. We used the findings of our previous studies for designing the digital maternity record^37,60^.

### Study procedure and schedules

Participants in the SMART Start study had the possibility to choose their level of involvement. Upon inclusion via the UKER, participants could opt-in for the digital maternity record package. Additionally, they could select from two optional smart self-examination packages: the standard care kit, the innovative kit, or both combined. Participants also had the option not to select any package. Participants who chose a device package automatically received the digital maternity record package.

For the self-examinations with the smart technologies, the following procedure was recommended to the study participants: blood pressure should be measured on two days per week, 2–3 days apart, both in the morning and evening. The scale should be used once per week, preferably on the same day each week. The urine test should be conducted once per week, preferably on the same day each week. The sleep mat should be used every night if possible. The smartwatch should be worn as often as possible, especially at night and during the day.

The study included structured data collection through questionnaires administered at predefined “visits” according to the gestational week.

Questionnaires assessed anxiety-driven health-related internet use (CSS), mental health (EPDS), health economics, sleep apnea risk (MAPI), health behaviors specific to pregnancy (PHBS), physical activity (PPAQ), sleep quality (PSQI), and sociodemographic characteristics. The health economics questionnaire has been developed by the Department of Health Management, Institute of Management of the Friedrich-Alexander-Universität Erlangen-Nürnberg.

After study inclusion, the first visit was the baseline visit. Subsequent visits were scheduled depending on gestational age at inclusion and labeled as ‘V’ followed by the gestational week (e.g., ‘V12’ for questionnaires distributed in weeks 10–11 to capture data by week 12. After delivery, the birth visit was conducted, with the postpartum visit taking place six weeks later.

Participants were given a two-week period to complete and submit each questionnaire. Duplicate questionnaires from the baseline visit and the following visit were only collected once. Birth-related questionnaires were available as soon as a birth is reported in the app, postpartum questionnaires six weeks later. Table 7 shows the detailed visits schedule of the questionnaires across pregnancy and the postpartum phase.

**Table 7:** Schedule of questionnaire administration across pregnancy and postpartum phases in alphabetic order

		I	II				III			
	BL	V12	V16	V20	V24	V28	V32	V36	Birth	PP
Cyberchondria Severity Scale (CSS)	x		x		x		x		x	
Edinburgh Postnatal Depression Scale (EPDS)								x	x	x
Health economy questionnaire	x							x		
Multivariable Apnea Prediction Index (MAPI)									x	
Pregnancy-related Health Behavior Scale (PHBS)	x						x			
Pregnancy Physical Activity Questionnaire (PPAQ)	x		x		x		x			
Pittsburgh Sleep Quality Index (PSQI)	x	x		x		x		x		
Sociodemographics questionnaire	x									

Questionnaire distribution is organized as visits according to gestational age. ‘BL’ denotes the baseline visit, conducted at study enrollment. Subsequent visits are labeled as ‘V’ followed by the gestational week (e.g., ‘V12’ for questionnaires distributed in weeks 10–11 to capture data by week 12). ‘PP’ indicates the postpartum phase. The trimesters are marked in Roman numerals (I, II, III).

### Data exclusion and imputation

In total, *n* = 528 participants have been included in the study. For analyses based on gestational age, participants were excluded if they either did not provide their gestational age or if the reported gestational age was implausible (e.g., conception date later than the study start date). This was the case for *n* = 166 participants.

To determine whether participants were still active in the study or had completed participation due to childbirth, individuals were excluded if they did not provide a birth date for the child or if the reported birth date was implausible (*n* = 443).

It was not possible to determine a specific study end date for all participants. Each participant had an individual study end date, which was typically defined as two weeks after the postpartum phase, i.e., 8 weeks after delivery, allowing participants sufficient time to complete postpartum questionnaires. However, app functionalities remained available beyond this point. For certain analyses, it was important to estimate how many participants were theoretically still active in the study at a given time to assess the retention probability. Therefore, an individual study end date was determined, defined as either eight weeks after the child’s reported birth date (if available) or eight weeks after week 40 of gestation if the actual birth date was not reported.

### Analysis and metrics

Gestational age has been provided by participants in the entry questionnaire. Based on the gestational age (week + day) the trimester was computed according to the guidelines of the German Society of Gynecology and Obstetrics (DGGG - Deutsche Gesellschaft für Gynäkologie und Geburtshilfe^61^): weeks 0 + 1–11 + 6: 1^st^ trimester, weeks 12 + 0–27 + 6: 2nd trimester, weeks 28 + 0–40 + 0: 3rd trimester.

We analyzed the usage behavior of the SMART Start app. As basis we used user interactions, defined as a single action or click within the app. A page view was defined as a sequence of interactions within the same page, with a maximum of 30 min between two consecutive interactions. A session was defined as a sequence of page views with no more than 30 min of inactivity between consecutive page views. We chose this threshold to accommodate the app’s longest expected content duration, specifically a 26 min MBSR audio file.

App pages were summarized by content types, e.g., “App setup” includes Withings device setup or calendar settings, “App settings” summarizes profile-related functions, such as password change, the option to retract consent or to change the color theme of the app. “Maternity record” included the three pages of the digital maternity record (previous pregnancies, gravidogram, pregnancy card). “Photo upload” summarized maternity record and urinalysis photo uploads, “Legal” combined the pages imprint, privacy and terms of use.

We analyzed data from the smart self-examination technologies across the following categories: sleep episodes, blood pressure measurements, urinalyses, ECG measurements, weight measurements, workouts, and activity days. For each category, we assessed the total number of transmitted data points per participant and the number of participants contributing data.

Sleep data were collected via the Withings ScanWatch and the Withings Sleep Analyzer and includes whole sleep nights as well as naps and interrupted periods of sleep.

Blood pressure (systolic/diastolic) was recorded using the Withings BPM Core device, and weight via the Withings Body+ scale.

Urinalysis data refer to completed app-based walkthroughs, each consisting of three pictures of reference card and test strip and five self-assessed urine parameters (protein, pH-value, glucose, nitrite, leucocytes).

ECG measurements from the Withings ScanWatch consisted of 30 s 1-lead recordings.

Workouts tracked with the Withings ScanWatch were considered if they lasted 10 min.

Activity days are defined as days, when the Withings ScanWatch was worn, and step count data have been recorded.

To evaluate participants’ interaction with the digital questionnaires and questionnaire schedules, we calculated two metrics: view-to-completion rate and scheduled completion rate.

The view-to-completion rate was defined as the ratio of submitted questionnaires to questionnaire page views. This measure reflects the proportion of questionnaire views that resulted in an actual submission, providing an estimate of participant engagement as well as the usability or perceived relevance of the questionnaire. Rates were calculated for each questionnaire as an overall percentage, and additionally on a per-participant level, summarized using the median and IQR.

The scheduled completion rate was defined as the ratio of submitted questionnaires to the number of scheduled submissions as determined by the study protocol. This metric provides an estimate of adherence to the intended study schedule. The expected number of submissions per questionnaire was derived individually for each participant based on their gestational age at study entry and the questionnaire schedule, see Table 7. Total rates were computed per questionnaire, and participant-level rates were summarized using median and IQR.

To evaluate participants’ adherence to the recommended self-examination schedules, we calculated the weekly protocol adherence rate. The weekly protocol adherence rate was defined as the ratio of study weeks in which a participant fully adhered to the recommended measurement schedule to the total number of study weeks. This metric was calculated separately for each self-examination type for which instructions were provided: blood pressure (twice per week), weight (once per week), urinalysis (once per week), and sleep (daily). For each week of participation, adherence was considered fulfilled if the respective measurement frequency was met in accordance with the study protocol. Participant-level adherence rates were summarized using median and interquartile range (IQR).

### Inferential statistics

To evaluate differences in app content usage across pregnancy, we conducted a two-way repeated-measures ANOVA with Trimester and content type as within-subject factors. Only participants with app activity recorded in all three trimesters were included. The dependent variable was the number of page views per content type and trimester. Sphericity was tested using Mauchly’s test where applicable, and Greenhouse-Geisser correction was applied where necessary. Effect sizes were reported using generalized eta squared (η^2^_G_).

Subgroups were defined based on self-reported participant characteristics: (1) subjective ease of technology use (high: “very easy” or “easy”; low: “neutral”, “hard”, or “very hard”), (2) interest in technology (high: “very interested” or “interested”; low: “neutral”, “not interested”, or “not interested at all”), (3) presence of children in the household (no children vs. ≥1 child), and (4) highest school education (high: high school diploma; low: General Certificate of Secondary Education, Certificate of Secondary Education, or no degree).

We performed survival analysis to assess participant retention over time using Kaplan-Meier curves. Comparator groups of interest were statistically evaluated using the log-rank test to identify significant differences in study dropout. The individual study end date was defined as eight weeks postpartum (based on the self-reported birth date if available or estimated as eight weeks after gestational week 40 otherwise). Participants were censored if their last completed study task occurred within this pre-specified study period.

We compared the subgroups in wearable data sharing, weekly protocol adherence rates, and scheduled completion rates, with Mann–Whitney U tests, as data were non-normally distributed. Effect sizes were reported using rank-biserial correlation coefficients. No correction for multiple comparisons was applied due to the exploratory nature of the subgroup analysis.

Statistical significance was defined as *p* ≤ 0.05. All analyses were conducted using Python (version 3.9).

## Supplementary information

supplementary_material_TC2

## Data Availability

The data that support the findings of this study are not openly available due to reasons of sensitivity to protect the study participants privacy. The data are available from the corresponding author upon reasonable request. Data are located in controlled access data storage at the Universitätsklinikum Erlangen.
