# Supplementary material for: Adherence to digital pregnancy care – lessons learned from the SMART start feasibility study
Source: NPJ Digit Med. 2025 Aug 30;8:561. doi: 10.1038/s41746-025-01966-8 (PMC12398578; doi:10.1038/s41746-025-01966-8)
Supplement: Supplementary file 1 — supplementary_material_TC2 [file 41746_2025_1966_MOESM1_ESM.pdf]

Supplementary Information

Supplementary Information ... *1*

Supplementary Figures ... **2**

Supplementary Figure 1: Overview of Participant Distribution over Study Visits ... 2

Supplementary Figure 2: In-App Workflow ... 3

Supplementary Figure 3: Screenshots SMART Start App - English-Language Version - User Engagement Enhancement and Informative Content ... 4

Supplementary Figure 4: Screenshots SMART Start App – English-Language Version - Self-Examination Overview ... 5

Supplementary Figure 5: Screenshots SMART Start App – English-Language Version - Urinalysis ... 6

Supplementary Figure 6: Screenshots SMART Start App – English-Language Version- Maternity Record . 7

Supplementary Figure 7: Screenshots SMART Start App - German-Language Original - User Engagement Enhancement and Informative Content ... 8

Supplementary Figure 8: Screenshots SMART Start App – German-Language Original - Self-Examination Overview ... 9

Supplementary Figure 9: Screenshots SMART Start App – German-Language Original - Urinalysis ... 10

Supplementary Figure 10: Screenshots SMART Start App – German-Language Original - Maternity Record ... 11

Supplementary Tables ... **12**

Supplementary Table 1: Overview of App Page Views ... 12

Supplementary Table 2: Page Views across Trimesters – ANOVA Results ... 13

Supplementary Table 3: Page Views across Trimesters – Post hoc Results ... 14

Supplementary Table 4: Wearable Data Sharing across Participant Subgroups ... 14

Supplementary Table 5: Adherence Patterns across Participant Subgroups ... 15

CONSORT 2010 Checklist of information to include when reporting a pilot or feasibility trial ... **17**

Supplementary Figures

Supplementary Figure 1: Overview of Participant Distribution over Study Visits

Number of participants that are able to participate in study visits based on the gestational age at study start. The first study visit V12 takes places in pregnancy weeks 10-11, the last study visit V36 takes place in pregnancy weeks 34-35. Participants that were included in the study after week 35 are not able to participate in the study visits. Absolute *participant counts are shown on top of the bars. The plot includes only participants that shared their pregnancy week (n=362)*. In total 319 participants were able to participate in study visit V36, which included pregnancy weeks 34-35, whereas only 48 participants have been recruited early enough to conduct V12 (pregnancy week 10-11). 43 participants have been included after week 35 of pregnancy and could, therefore, not participate in visits before delivery.

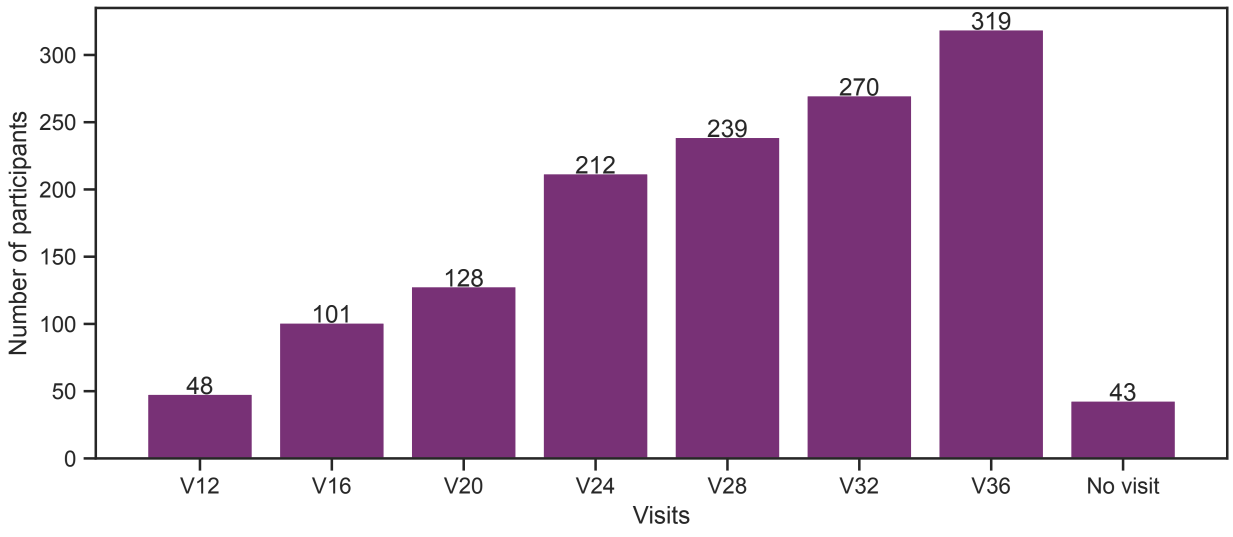

Supplementary Figure 2: In-App Workflow

User flowchart illustrating the in-app workflow of a user journey in detail.

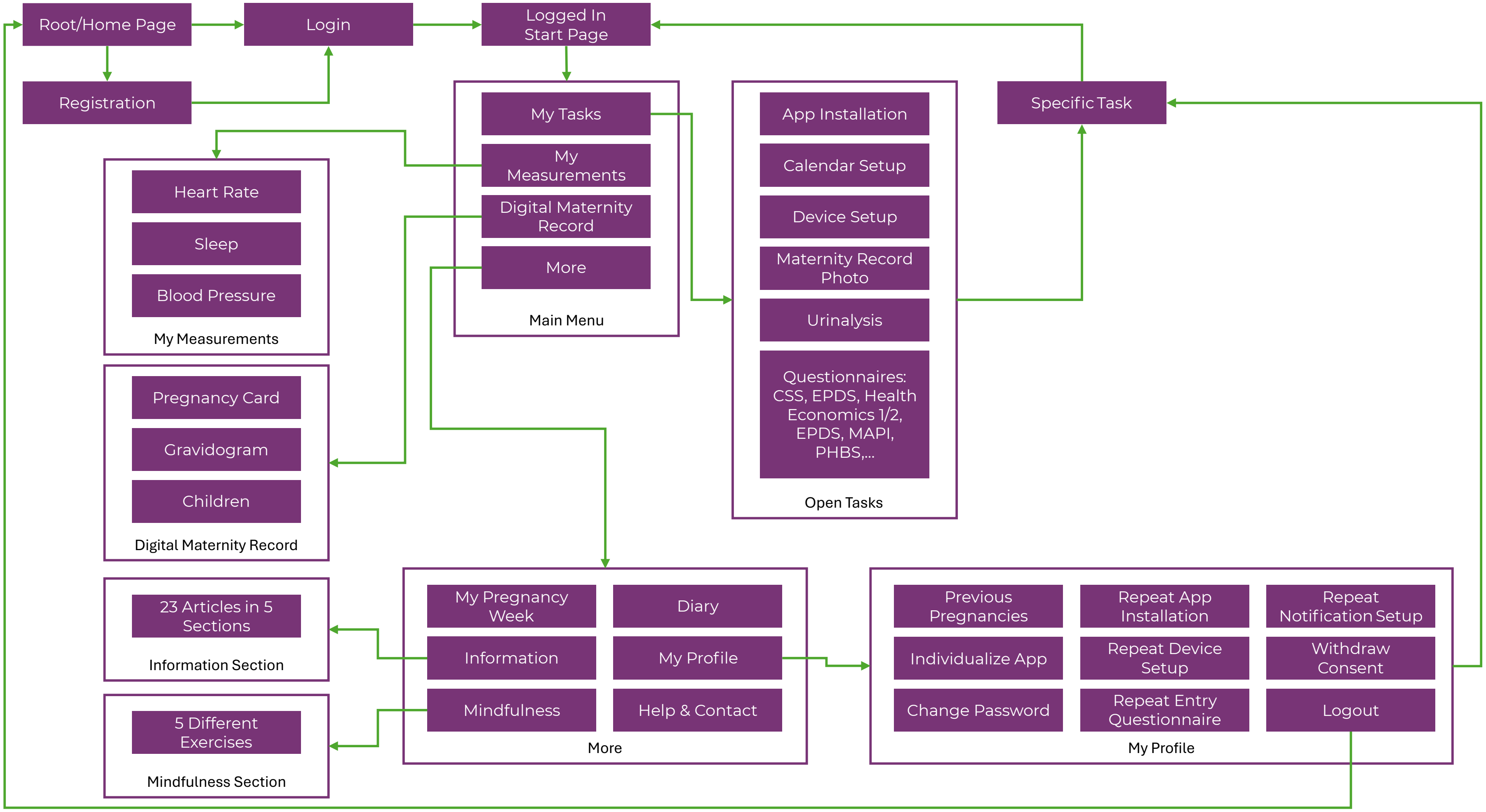

Supplementary Figure 3: Screenshots SMART Start App - English-Language Version - User Engagement Enhancement and Informative Content

Screenshots showing SMART Start app pages designed to enhance user engagement and provide informative content. Upper left: Information section containing material regarding pregnancy, delivery and postpartum, upper middle: audio exercises for mindfulness-based stress reduction, upper right: Personalized information section depending on gestational week, lower left: journal function to track emotional state, lower right: tasks overview page providing a summary of pending tasks.

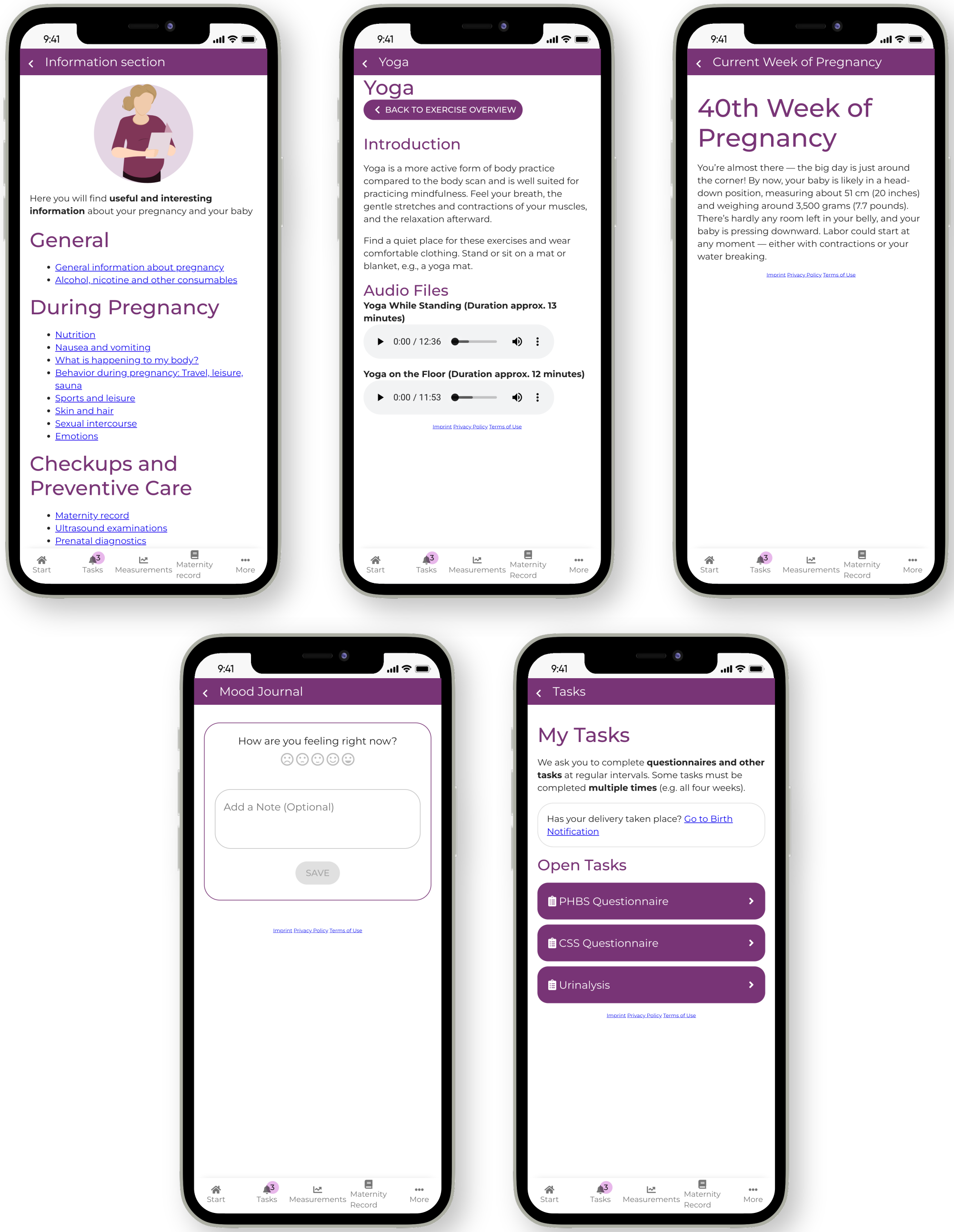

Supplementary Figure 4: Screenshots SMART Start App – English-Language Version - Self-Examination Overview

Screenshots showing the SMART Start app pages for self-examination measurements. Left: Overview page for heart rate, sleep and blood pressure, right: measurement values for heart rate in a weekly overview.

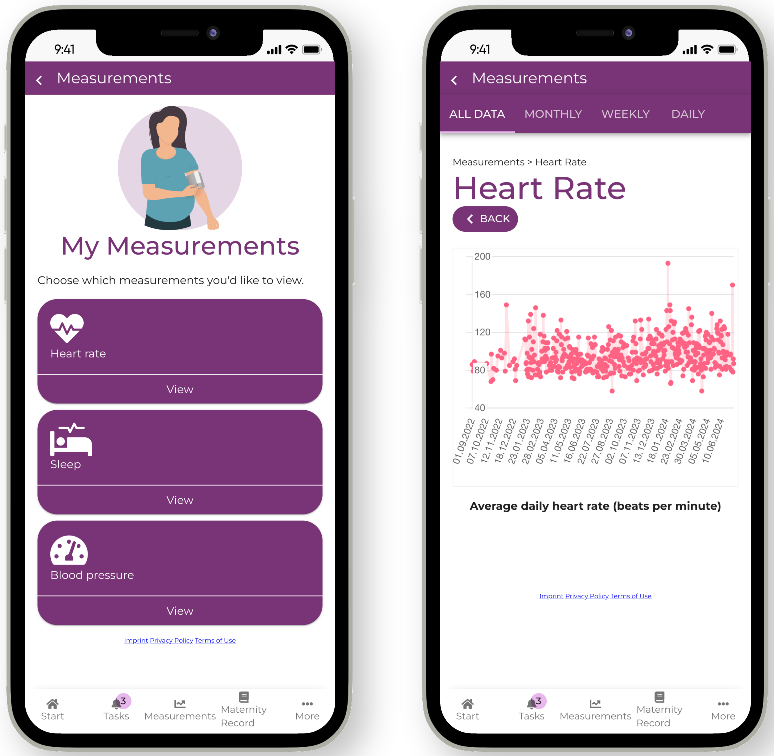

Supplementary Figure 5: Screenshots SMART Start App – English-Language Version - Urinalysis

Screenshots of the step-by-step instructions to self-performed urinalysis. Left: Introduction into the app-based urinalysis, Middle: Dip stick into urine, Right: Position dip stick next to reference card and create good conditions for photo.

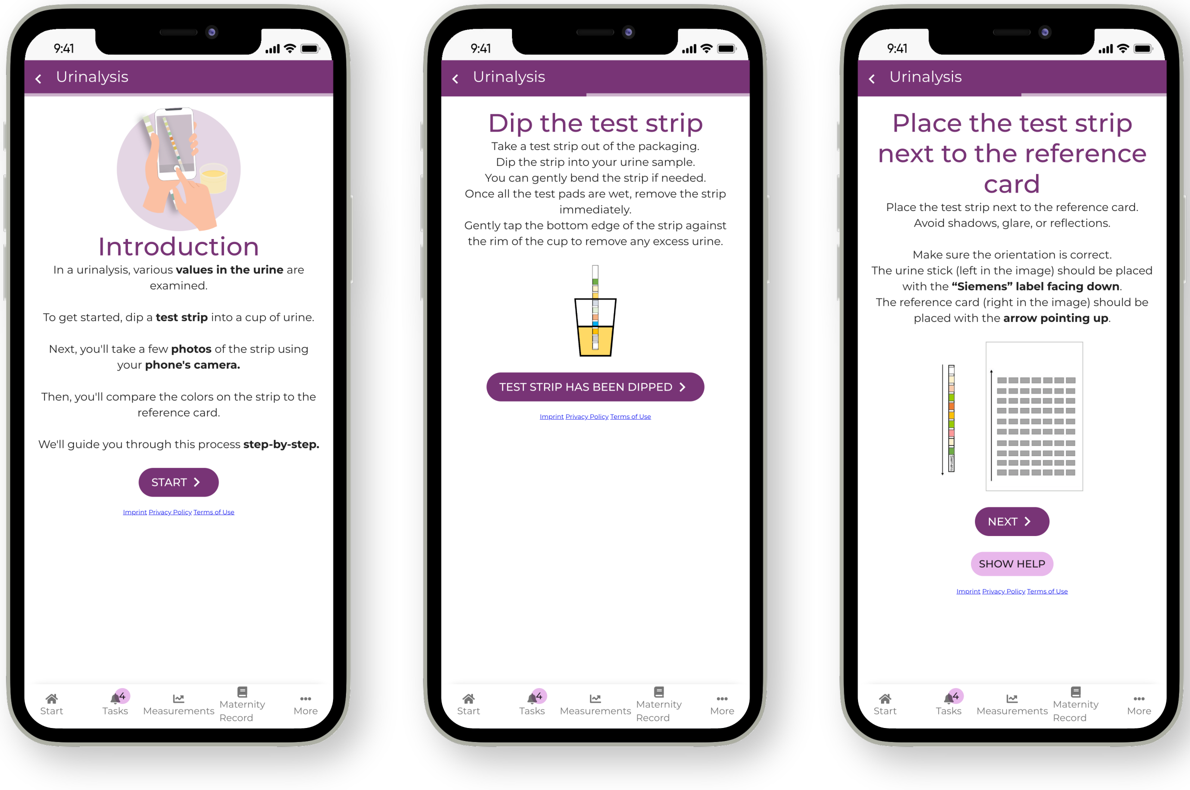

Supplementary Figure 6: Screenshots SMART Start App – English-Language Version-Maternity Record

Screenshot of the digital maternity record section in the SMART Start app. Left: content overview, right: pregnancy card with relevant information in case of an emergency.

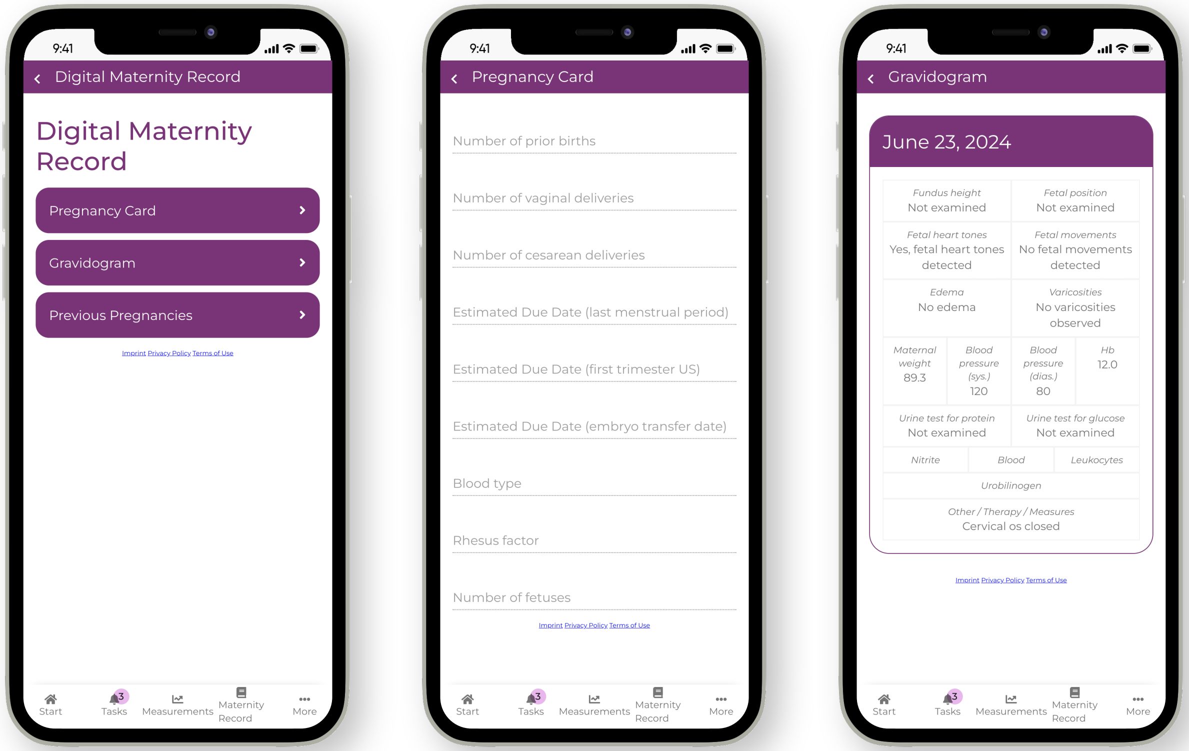

Supplementary Figure 7: Screenshots SMART Start App - German-Language Original - User Engagement Enhancement and Informative Content

Screenshots showing SMART Start app pages designed to enhance user engagement and provide informative content in German-language original. Upper left: Information section containing material regarding pregnancy, delivery and postpartum, upper middle: audio exercises for mindfulness-based stress reduction, upper right: Personalized information section depending on gestational week, lower left: journal function to track emotional state, lower right: tasks overview page providing a summary of pending tasks.

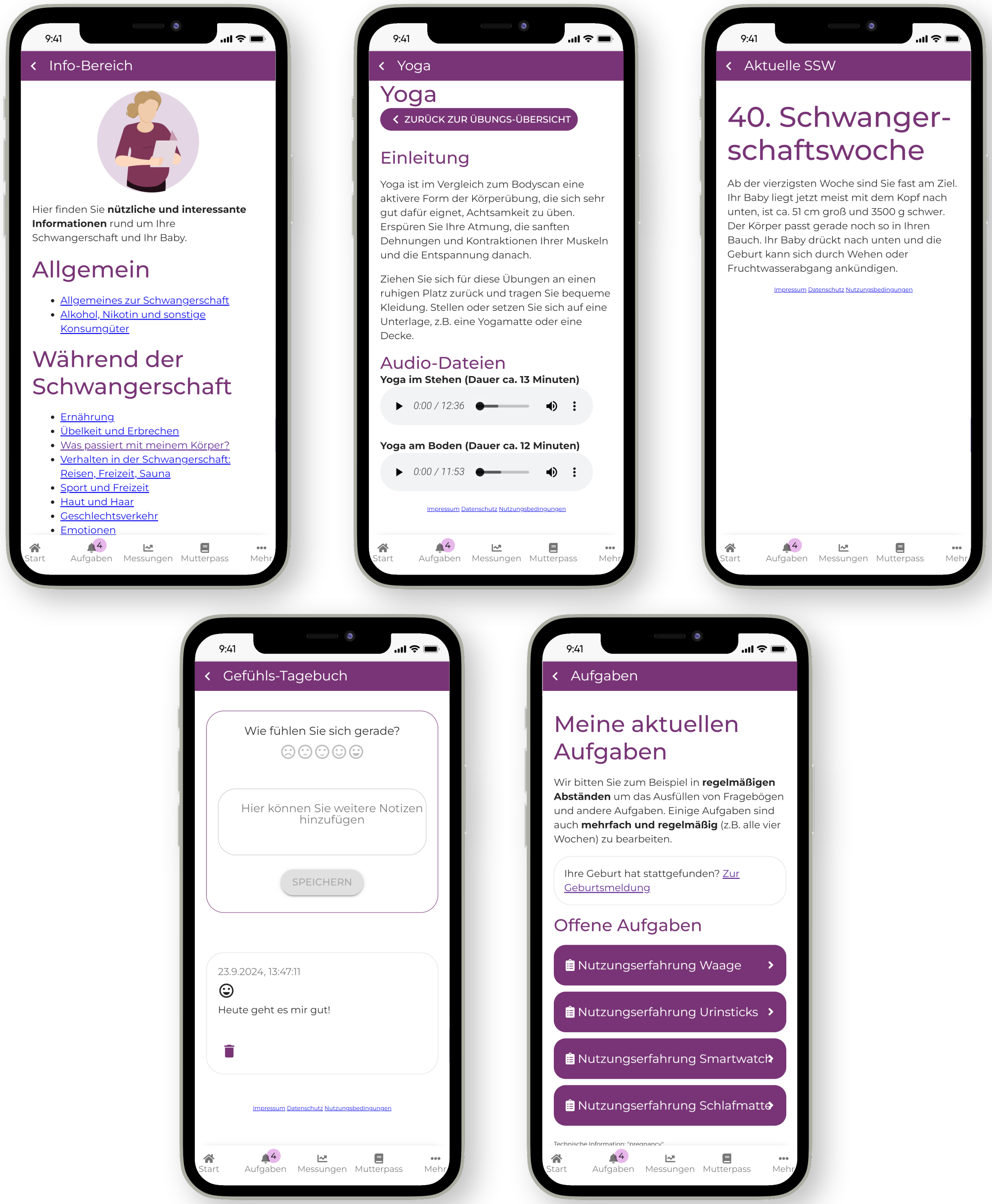

Supplementary Figure 8: Screenshots SMART Start App – German-Language Original - Self-Examination Overview

Screenshots showing the SMART Start app pages for self-examination measurements in German-language original. Left: Overview page for heart rate, sleep and blood pressure, right: measurement values for heart rate in a weekly overview.

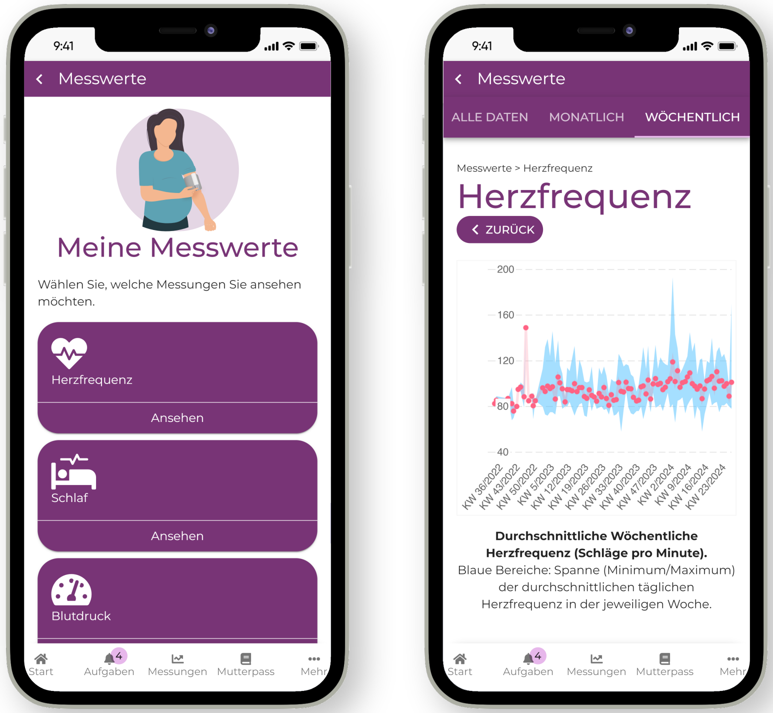

Supplementary Figure 9: Screenshots SMART Start App – German-Language Original - Urinalysis

Screenshots of the step-by-step instructions to self-performed urinalysis in German-language original. Left: Introduction into the app-based urinalysis, Middle: Dip stick into urine, Right: Position dip stick next to reference card and create good conditions for photo.

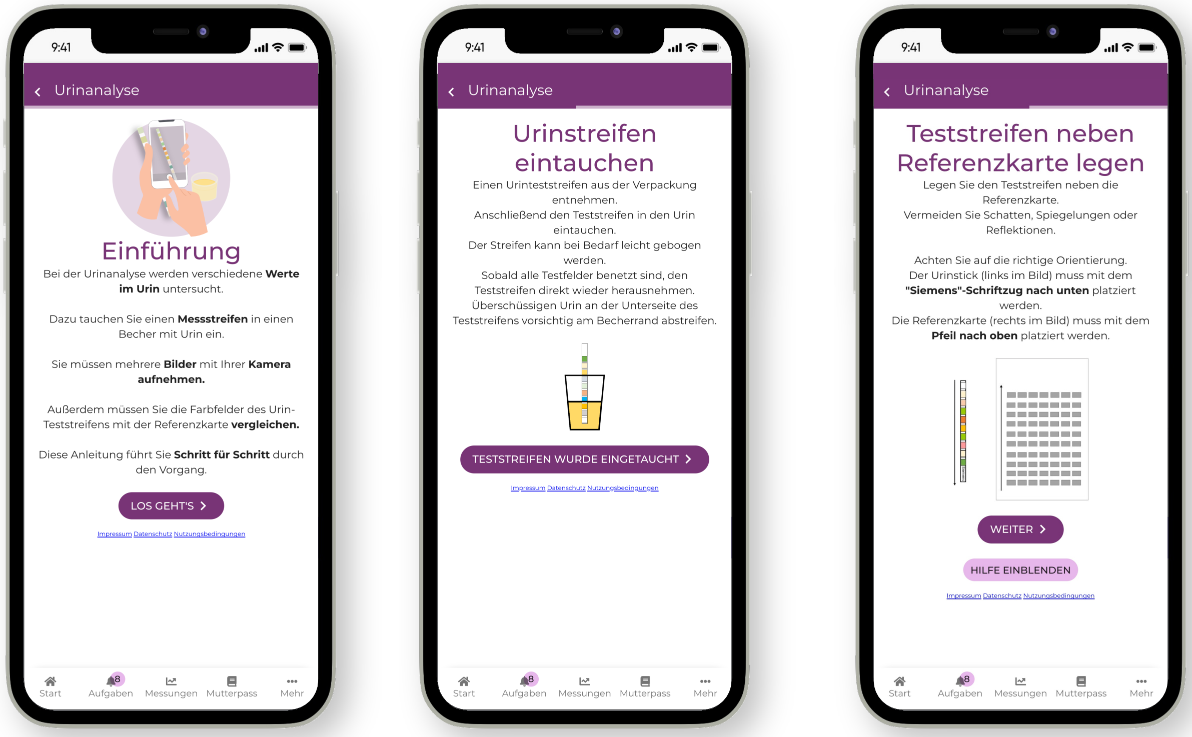

Supplementary Figure 10: Screenshots SMART Start App – German-Language Original - Maternity Record

Screenshot of the digital maternity record section in the SMART Start app in German-language original. Left: content overview, right: pregnancy card with relevant information in case of an emergency.

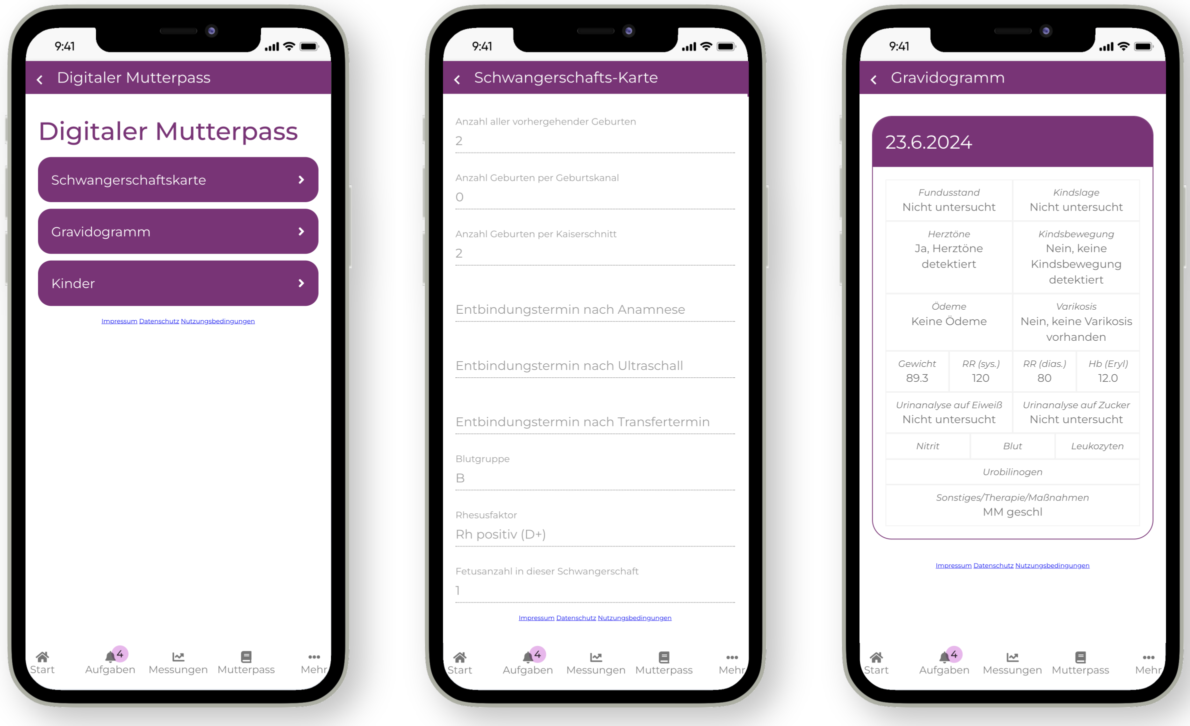

Supplementary Tables

Supplementary Table 1: Overview of App Page Views

Overview of app page views by content type, showing total page views, page view duration, session starts and ends. A session is defined as a sequence of page views that have no more than 30 min in between two consecutive pages. Session start is the first interacted page of a session, session end the last. Information pages, maternity record pages and questionnaires, and wearable overview pages are described separately. Characteristics are reported as either 1) the percentage of the total number of page views (n= 62,404), or 2) median value with 25th and 75th percentile, or 3) the percentage of the total number of sessions defined (n=5,984). The *Start page* has the most page views (24.9%), followed by *Tasks* (20.8%) and *Questionnaires* (8.7%). Least interacted page groups are *Journal*, *Help* and *Legal* representing <1% of all page views. The median duration of a page view varied across app pages, with the longest durations observed for *Photo uploads* with 190 seconds (IQR 14–325), followed by the *Pregnancy Physical Activity Questionnaire (PPAQ)* with 196 seconds (IQR 54–271) and the *Pittsburgh Sleep Quality Index (PSQI)* with 167 seconds (IQR 26–246).

	**Page views, n (%)**	**Page view duration in seconds, median (IQR)**	**Session starts, n (%)**	**Session ends, n (%)**
App settings	3,732 (6.0)	3 (2-5)	21 (0.3)	258 (3.7)
App setup	2,199 (3.5)	23 (9-50)	20 (0.3)	100 (1.5)
Help	128 (0.2)	8 (4-32)	0 (0.0)	39 (0.6)
Information pages	2,123 (3.4)	9 (4-43)	10 (0.1)	164 (2.4)
Birth	220 (0.4)	105 (30-196)	0 (0.0)	25 (0.4)
During pregnancy	282 (0.4)	82 (33-157)	1 (0.0)	40 (0.6)
General	31 (0.0)	63 (29-158)	0 (0.0)	1 (0.0)
Overview	1331 (2.1)	5 (3-10)	8 (0.1)	70 (1.0)
Perinatal	179 (0.3)	46 (15-96)	1 (0.0)	16 (0.2)
Puerpetal	80 (0.1)	146 (29-252)	0 (0.0)	12 (0.2)
Journal	137 (0.2)	3 (2-7)	1 (0.0)	3 (0.0)
Legal	91 (0.1)	4 (2-9)	0 (0.0)	3 (0.0)
Maternity record	5,219 (8.4)	2 (1-4)	23 (0.3)	338 (4.9)
Overview	3,362 (5.4)	2 (1-3)	20 (0.3)	188 (2.7)
Previous pregnancies	384 (0.6)	2 (1-3)	0 (0.0)	9 (0.1)
Gravidogram	691 (1.1)	7 (2-33)	2 (0.0)	113 (1.6)
Pregnancy card	782 (1.3)	5 (3-11)	1 (0.0)	28 (0.4)
Mindfulness	992 (1.6)	4 (2-8)	8 (0.1)	99 (1.4)
My pregnancy week	2,360 (3.8)	13 (4-24)	16 (0.2)	470 (6.8)
Photo upload	1,611 (2.6)	190 (14-325)	34 (0.5)	148 (2.1)
Questionnaires	5,428 (8.7)	80 (15-159)	46 (0.7)	241 (3.5)

Cyberchondria Severity Scale (CSS)	715 (1.1)	105 (67-141)	3 (0.0)	27 (0.4)
Edinburgh Postnatal Depression Scale (EPDS)	308 (0.5)	56 (14-81)	7 (0.1)	8 (0.1)
Multivariable Apnea Prediction Index (MAPI)	92 (0.1)	23 (17-33)	0 (0.0)	1 (0.0)
Pregnancy-related Health Behavior Scale (PHBS)	390 (0.6)	89 (69-119)	2 (0.0)	10 (0.1)
Pregnancy Physical Activity Questionnaire (PPAQ)	644 (1.0)	196 (54-271)	6 (0.1)	26 (0.4)
Pittsburgh Sleep Quality Index (PSQI)	705 (1.1)	167 (26-246)	2 (0.0)	53 (0.8)
Social demographics	288 (0.5)	82 (65-113)	2 (0.0)	3 (0.0)
Health economy	687 (1.1)	13 (5-233)	16 (0.2)	69 (1.0)
Usability	1,074 (1.7)	67 (43-96)	6 (0.1)	34 (0.5)
Misc	523 (0.8)	31 (9-68)	2 (0.0)	10 (0.1)
Start page	15,534 (24.9)	3 (2-6)	6,481 (94.1)	3,207 (46.6)
Tasks	12,999 (20.8)	2 (1-5)	154 (2.2)	1,149 (16.7)
Wearable overview	9,851 (15.8)	2 (2-6)	71 (1.0)	666 (9.7)
Overview	6,267 (10.1)	2 (1-3)	62 (0.9)	407 (5.9)
Blood pressure	1,176 (1.9)	5 (2-11)	3 (0.0)	127 (1.8)
Heart rate	1,344 (2.2)	6 (3-13)	6 (0.1)	48 (0.7)
Sleep	1,064 (1.7)	6 (2-14)	0 (0.0)	84 (1.2)

Supplementary Table 2: Page Views across Trimesters – ANOVA Results

Results of the two-way repeated-measures ANOVA testing the main effects of Trimester and content type, and their interaction, on app page views. Greenhouse-Geisser corrected p-values and generalized eta squared (η^2_G) are reported. Only participants with app activity recorded in all three trimesters were included. The dependent variable was the number of page views per content type and trimester. Sphericity was tested using Mauchly's test where applicable, and Greenhouse-Geisser correction was applied where necessary. Effect sizes were reported using generalized eta squared (η^2_G).

Effect	F	p-GG-corr	η^2_G
Trimester	9.21	0.0004	0.042
Content type	66.20	< 0.0001	0.392
Trimester * Content type	8.45	< 0.0001	0.095

Supplementary Table 3: Page Views across Trimesters – Post hoc Results

Post hoc pairwise comparisons between trimesters for app content types, using paired t-tests with Holm correction for multiple comparisons. Cohen's d indicates the effect size for each contrast. Only statistically significant results are shown.

Content type	Comparison	p-corr	Cohen's d
App setup	1st vs. 2nd	0.001	1.097
App setup	2nd vs. 3rd	0.001	0.840
App setup	1st vs. 3rd	< 0.001	2.200
Mindfulness	2nd vs. 3rd	0.001	0.775
Mindfulness	1st vs. 3rd	0.002	0.602
My pregnancy week	1st vs. 2nd	< 0.001	-0.787
My pregnancy week	2nd vs. 3rd	< 0.001	0.490
Photo upload	1st vs. 2nd	< 0.001	-1.417
Photo upload	1st vs. 3rd	< 0.001	-1.045
Start page	1st vs. 2nd	< 0.001	-0.997
Start page	2nd vs. 3rd	0.025	0.480
Tasks	1st vs. 2nd	0.004	-0.811
Wearable overview	1st vs. 2nd	0.012	-0.690
Wearable overview	2nd vs. 3rd	< 0.001	1.063

Supplementary Table 4: Wearable Data Sharing across Participant Subgroups

Summary of data collected from smart self-examinations technology usage across four self-reported participant characteristics: subjective ease of technology use, interest in technology, presence of children in the household, and highest school education. Data are presented as the total number of data points, the number of participants sharing each data type and the median (IQR) data points per participant. We examined whether the amount of data shared differed across participant subgroups. Mann-Whitney U tests for each subgroup–measurement combination revealed no significant differences. Detailed results are provided in the table.

	**Average number of shared datapoints per participant, median (IQR)**						
**Characteristic**	Blood pressure	Weight	Sleep	Urinalysis	Activity days	ECG	Workouts
Subjective ease of technology use							
Low (n=43)	13.0 (7.0–45.0)	24.0 (18.0–40.0)	366.0 (245.0–545.0)	5.0 (4.0–8.0)	125.0 (22.0–163.0)	14.0 (5.0–73.0)	166.0 (2.0–214.0)
High (n=194)	14.0 (4.0–70.0)	19.0 (2.0–40.0)	254.0 (2.0–441.0)	3.0 (0.0–8.0)	55.0 (5.0–120.0)	25.0 (4.0–93.0)	37.0 (0.0–181.0)
p-value	*p= 0.956*	*p = 0.216*	*p = 0.107*	*0.190*	*p = 0.107*	*p = 0.667*	*p = 0.403*
Interest in technology							
Low (n=134)	13.0	20.0	288.0	3.0	94.0	14.0	37.0

	(4.0–47.0)	(7.0–36.0)	(10.0–466.0)	(0.0–8.0)	(14.0–156.0)	(3.0–73.0)	(2.0–177.0)
High (n=103)	27.0 (11.0–72.0)	19.0 (1.0–65.0)	254.0 (19.0–489.0)	5.0 (1.0–9.0)	67.0 (6.0–120.0)	30.0 (11.0–93.0)	103.0 (3.0–221.0)
p-value	*p= 0.260*	*p = 0.873*	*p = 0.927*	*p = 0.251*	*p = 0.873*	*p = 0.214*	*p = 0.456*
Children in household							
No children (n= 134)	15.0 (10.0–69.0)	19.5 (13.2–39.0)	328.0 (56.0–523.5)	4.0 (1.0–8.0)	80.5 (16.2–120.0)	27.0 (10.2–97.5)	64.0 (2.2–160.2)
≥1 children (n=103)	13.0 (2.8–65.2)	20.5 (2.8–45.0)	251.0 (2.5–337.5)	2.0 (0.0–6.0)	62.5 (9.8–212.8)	4.5 (0.8–62.5)	53.0 (1.5–201.8)
p-value	*p = 0.612*	*p = 0.880*	*p = 0.380*	*p = 0.301*	*p = 0.474*	*p = 0.083*	*p = 0.825*
Highest school education							
Low (n=75)	43.5 (8.5–73.5)	19.5 (8.8–42.2)	306.0 (0.0–464.5)	4.5 (0.0–8.0)	102.5 (0.8–121.2)	25.0 (4.8–96.5)	42.5 (0.0–112.0)
High (n=162)	12.5 (5.5–59.8)	20.0 (4.8–39.0)	257.5 (26.0–478.8)	3.0 (0.2–9.0)	54.0 (16.2–152.0)	17.5 (4.0–81.5)	83.5 (3.0–214.5)
p-value	*p= 0.556*	*p = 0.781*	*p = 0.627*	*p = 0.906*	*p = 0.746*	*p = 0.917*	*p = 0.443*

Supplementary Table 5: Adherence Patterns across Participant Subgroups

Scheduled completion rate per participant for individual questionnaires (submitted questionnaires / scheduled submissions) across four self-reported participant characteristics: subjective ease of technology use, interest in technology, presence of children in the household, and highest school education. Adherence rate is reported as median and interquartile range (IQR) in %. Group comparisons were conducted using Mann-Whitney U tests. Statistical significance is indicated as * p≤0.05, **p≤0.01, ***p≤0.001. Participants without children showed significantly higher completion rates for the EPDS (16.7% vs. 0.0%, $p < 0.05$) and PSQI (66.7% vs. 50.0%, $p < 0.05$) compared to those with children. Higher educational attainment was associated with higher EPDS completion rates (0.0–66.7% vs. 0.0–33.3%, $p < 0.05$). No significant differences in scheduled questionnaire completion rates were found with respect to subjective ease of technology use or interest in technology. The MAPI, PHBS, PPAQ, CSS, sociodemographic questionnaire, and health economy questionnaire were completed at similarly high rates across all participant subgroups, regardless of technological affinity, household composition, or educational background.

	**Scheduled completion rate per participant, median (IQR) in %**			
**Characteristic**	Edinburgh Postnatal Depression Scale (EPDS)	Multivariable Apnea Prediction Index (MAPI)	Pregnancy-related Health Behavior Scale (PHBS)	Pregnancy Physical Activity Questionnaire (PPAQ)
Subjective ease of technology use				
Low (n=43)	0.0 (0.0–33.3)	0.0 (0.0–0.0)	50.0 (50.0–100.0)	66.7 (33.3–100.0)
High (n=194)	0.0 (0.0–62.5)	0.0 (0.0–100.0)	100.0 (50.0–100.0)	66.7 (33.3–100.0)
P-value	*p = 0.092*	*p = 0.138*	*p = 0.241*	*p = 0.269*
Interest in technology				

Low (n=134)	0.0 (0.0–66.7)	0.0 (0.0–100.0)	100.0 (50.0–100.0)	66.7 (33.3–100.0)
High (n=103)	0.0 (0.0–33.3)	0.0 (0.0–100.0)	50.0 (50.0–100.0)	66.7 (33.3–100.0)
P-value	*p = 0.911*	*p = 0.803*	*p = 0.222*	*p = 0.568*
Children in household				
No children (n= 134)	16.7 (0.0–66.7)*	0.0 (0.0–100.0)	100.0 (50.0–100.0)	66.7 (50.0–100.0)
≥1 children (n=103)	0.0 (0.0–33.3)*	0.0 (0.0–100.0)	50.0 (50.0–100.0)	66.7 (33.3–100.0)
P-value	*p = 0.041*	*p = 0.101*	*p = 0.546*	*p = 0.146*
Highest school education				
Low (n=75)	0.0 (0.0–33.3)*	0.0 (0.0–50.0)	100.0 (50.0–100.0)	66.7 (33.3–100.0)
High (n=162)	0.0 (0.0–66.7)*	0.0 (0.0–100.0)	100.0 (50.0–100.0)	66.7 (33.3–100.0)
P-value	*p = 0.043*	*p = 0.092*	*p = 0.729*	*p = 0.960*
	**Scheduled completion rate per participant, median (IQR) in %**			
**Characteristic**	Pittsburgh Sleep Quality Index (PSQI)	Cyberchondria Severity Scale (CSS)	Sociodemographics questionnaire	Health economy questionnaire
Subjective ease of technology use				
Low (n=43)	50.0 (33.3–100.0)	50.0 (25.0–75.0)	100.0 (100.0–100.0)	50.0 (50.0–100.0)
High (n=194)	55.0 (33.3–100.0)	50.0 (33.3–100.0)	100.0 (100.0–100.0)	50.0 (50.0–100.0)
P-value	*p = 0.199*	*p = 0.177*	*p = 1.000*	*p = 0.086*
Interest in technology				
Low (n=134)	50.0 (33.3–100.0)	50.0 (33.3–100.0)	100.0 (100.0–100.0)	50.0 (50.0–100.0)
High (n=103)	60.0 (33.3–100.0)	50.0 (29.2–80.0)	100.0 (100.0–100.0)	50.0 (50.0–100.0)
P-value	*p = 0.933*	*p = 0.674*	*p = 1.000*	*p = 0.446*
Children in household				
No children (n= 134)	66.7 (33.3–100.0)*	55.0 (40.0–80.0)	100.0 (100.0–100.0)	50.0 (50.0–100.0)
≥1 children (n=103)	50.0 (33.3–100.0)*	50.0 (25.0–100.0)	100.0 (100.0–100.0)	50.0 (50.0–100.0)
P-value	*p = 0.043*	*p = 0.059*	*p = 1.000*	*p = 0.846*
Highest school education				
Low (n=75)	50.0 (33.3–100.0)	50.0 (25.0–70.8)	100.0 (100.0–100.0)	50.0 (50.0–100.0)
High (n=162)	50.0 (33.3–100.0)	50.0 (33.3–100.0)	100.0 (100.0–100.0)	50.0 (50.0–100.0)
P-value	*p = 0.571*	*p = 0.133*	*p = 1.000*	*p = 0.245*

CONSORT 2010 Checklist of information to include when reporting a pilot or feasibility trial

CONSORT 2010 checklist of information to include when reporting a pilot or feasibility trial*

Section/Topic	Item No	Checklist item	Reported on page No
**Title and abstract**			
	1a	Identification as a pilot or feasibility randomised trial in the title	p. 1
	1b	Structured summary of pilot trial design, methods, results, and conclusions (for specific guidance see CONSORT abstract extension for pilot trials)	p. 1
**Introduction**			
Background and objectives	2a	Scientific background and explanation of rationale for future definitive trial, and reasons for randomised pilot trial	pp. 2-4
	2b	Specific objectives or research questions for pilot trial	pp.3-4
**Methods**			
Trial design	3a	Description of pilot trial design (such as parallel, factorial) including allocation ratio	pp.23-24
	3b	Important changes to methods after pilot trial commencement (such as eligibility criteria), with reasons	N/A – no changes reported
Participants	4a	Eligibility criteria for participants	p. 26
	4b	Settings and locations where the data were collected	pp. 23-26
	4c	How participants were identified and consented	p. 26
Interventions	5	The interventions for each group with sufficient details to allow replication, including how and when they were actually administered	pp. 23-24, p. 29
Outcomes	6a	Completely defined prespecified assessments or measurements to address each pilot trial objective specified in 2b, including how and when they were assessed	p. 29-32
	6b	Any changes to pilot trial assessments or measurements after the pilot trial commenced, with reasons	N/A
	6c	If applicable, prespecified criteria used to judge whether, or how, to proceed with future definitive trial	N/A
Sample size	7a	Rationale for numbers in the pilot trial	p. 24
	7b	When applicable, explanation of any interim analyses and stopping guidelines	N/A
Randomisation:			
Sequence generation	8a	Method used to generate the random allocation sequence	N/A no randomization
	8b	Type of randomisation(s); details of any restriction (such as blocking and block size)	N/A no

			randomization
Allocation concealment mechanism	9	Mechanism used to implement the random allocation sequence (such as sequentially numbered containers), describing any steps taken to conceal the sequence until interventions were assigned	N/A
Implementation	10	Who generated the random allocation sequence, who enrolled participants, and who assigned participants to interventions	N/A
Blinding	11a	If done, who was blinded after assignment to interventions (for example, participants, care providers, those assessing outcomes) and how	N/A
	11b	If relevant, description of the similarity of interventions	N/A
Statistical methods	12	Methods used to address each pilot trial objective whether qualitative or quantitative	p. 32
**Results**			
Participant flow (a diagram is strongly recommended)	13a	For each group, the numbers of participants who were approached and/or assessed for eligibility, randomly assigned, received intended treatment, and were assessed for each objective	p. 4 (Figure 1)
	13b	For each group, losses and exclusions after randomisation, together with reasons	p. 4
Recruitment	14a	Dates defining the periods of recruitment and follow-up	p. 23
	14b	Why the pilot trial ended or was stopped	p. 23, end of plannend recruitment period
Baseline data	15	A table showing baseline demographic and clinical characteristics for each group	pp. 5-8 (Tables 1,2)
Numbers analysed	16	For each objective, number of participants (denominator) included in each analysis. If relevant, these numbers should be by randomised group	throughout Results section pp. 4-16
Outcomes and estimation	17	For each objective, results including expressions of uncertainty (such as 95% confidence interval) for any estimates. If relevant, these results should be by randomised group	throughout Results section pp. 4-16
Ancillary analyses	18	Results of any other analyses performed that could be used to inform the future definitive trial	Results section and Supplementary Material

Harms	19	All important harms or unintended effects in each group (for specific guidance see CONSORT for harms)	N/A – no adverse events reported
	19a	If relevant, other important unintended consequences	N/A
**Discussion**			
Limitations	20	Pilot trial limitations, addressing sources of potential bias and remaining uncertainty about feasibility	in Discussion pp. 17-23
Generalisability	21	Generalisability (applicability) of pilot trial methods and findings to future definitive trial and other studies	in Discussion pp. 17-23
Interpretation	22	Interpretation consistent with pilot trial objectives and findings, balancing potential benefits and harms, and considering other relevant evidence	in Discussion pp. 17-23
	22a	Implications for progression from pilot to future definitive trial, including any proposed amendments	p. 23
**Other information**			
Registration	23	Registration number for pilot trial and name of trial registry	Abstract and p. 1 (DRKS00036 867)
Protocol	24	Where the pilot trial protocol can be accessed, if available	p. 1
Funding	25	Sources of funding and other support (such as supply of drugs), role of funders	p. 33
	26	Ethical approval or approval by research review committee, confirmed with reference number	p. 25

Citation: Eldridge SM, Chan CL, Campbell MJ, Bond CM, Hopewell S, Thabane L, et al. CONSORT 2010 statement: extension to randomised pilot and feasibility trials. BMJ. 2016;355. This is an Open Access article distributed in accordance with the terms of the Creative Commons Attribution (CC BY 3.0) license (http://creativecommons.org/licenses/by/3.0/), which permits others to distribute, remix, adapt and build upon this work, for commercial use, provided the original work is properly cited.

*We strongly recommend reading this statement in conjunction with the CONSORT 2010, extension to randomised pilot and feasibility trials, Explanation and Elaboration for important clarifications on all the items. If relevant, we also recommend reading CONSORT extensions for cluster randomised trials, non-inferiority and equivalence trials, non-pharmacological treatments, herbal interventions, and pragmatic trials. Additional extensions are forthcoming: for those and for up-to-date references relevant to this checklist, see www.consort-statement.org.
